# Animal Models and Integrated Nested Laplace Approximations

**DOI:** 10.1534/g3.113.006700

**Published:** 2013-08-01

**Authors:** Anna Marie Holand, Ingelin Steinsland, Sara Martino, Henrik Jensen

**Affiliations:** * Centre for Biodiversity Dynamics, Department of Biology, NTNU, NO-7491 Trondheim, Norway; † Centre for Biodiversity Dynamics, Department of Mathematical Sciences, NTNU, NO-7491 Trondheim, Norway

**Keywords:** additive genetic models, approximate Bayesian inference, heritability, quantitative genetics, AnimalINLA

## Abstract

Animal models are generalized linear mixed models used in evolutionary biology and animal breeding to identify the genetic part of traits. Integrated Nested Laplace Approximation (INLA) is a methodology for making fast, nonsampling-based Bayesian inference for hierarchical Gaussian Markov models. In this article, we demonstrate that the INLA methodology can be used for many versions of Bayesian animal models. We analyze animal models for both synthetic case studies and house sparrow (*Passer domesticus*) population case studies with Gaussian, binomial, and Poisson likelihoods using INLA. Inference results are compared with results using Markov Chain Monte Carlo methods. For model choice we use difference in deviance information criteria (DIC). We suggest and show how to evaluate differences in DIC by comparing them with sampling results from simulation studies. We also introduce an R package, AnimalINLA, for easy and fast inference for Bayesian Animal models using INLA.

To estimate the additive genetic variance (and thus the heritability) of different kinds of traits, biologists and animal breeders often use a generalized linear mixed model (GLMM), called an *animal model*. In an animal model individual *i*’s trait *y_i_* has a genetic part, *u_i_*. The value *u_i_* is known as the breeding value of individual *i*. From the assumption that the breeding value is the sum of effects of many genes and from the central limit theorem, the breeding values are assumed to have a Gaussian distribution with a dependence structure given by the pedigree. Since early 1980s, animal breeders have successfully used a frequentist approach with restricted maximum likelihood (REML), to for example increase meat or milk yield in cattle (Simm 1998). However, inference with REML is not trivial for GLMM models. Models for non-Gaussian traits especially are challenging in regard to uncertainty in breeding values and other parameter estimates (Tempelman and Gianola 1994; Sorensen and Gianola 2002; Bolker *et al.* 2009; Fong *et al.* 2010). A popular approach is best linear unbiased prediction (BLUP) (Henderson 1950) for calculating breeding values (Wilson *et al.* 2009). However, BLUP ignores all the uncertainty associated with the estimation and are not suitable for hypothesis testing in evolutionary questions (Postma 2006; Wilson *et al.* 2009; Hadfield *et al.* 2010). Another approach is to perform modeling in a Bayesian framework. All parameters are then considered random variables, and it is (in theory) straightforward to account for all uncertainty jointly in parameter estimates. This solves the problems making inference for non-Gaussian traits (Tempelman and Gianola 1994; Fong *et al.* 2010) and accounting for estimation uncertainty in the breeding values. Further, Bayesian modeling also solves many of the issues regarding analysis of breeding values discussed in Postma (2006), Wilson *et al.* (2009), and Hadfield *et al.* (2010) as both breeding values and functions of breeding values (*e.g.*, mean breeding values over hatch years) are considered random variables, and hence both uncertainty and dependencies are accounted for. This flexibility of the Bayesian framework has made Bayesian animal models increasingly popular. They have been used in animal breeding since early 1990s, whereas they only recently have been introduced to evolutionary biology (Kruuk *et al.* 2008; O’Hara *et al.* 2008; Ovaskainen *et al.* 2008; Hadfield 2010; Steinsland and Jensen 2010). See Gianola and Fernando (1986) and Sorensen and Gianola (2002) for a discussion of Bayesian animal models.

Except in a few special cases, Bayesian models do not have closed form analytic expression for quantities of typical interest, *e.g.*, posterior means. Hence, numerical approximations are needed. The traditional approximation procedure for Bayesian models is Markov Chain Monte Carlo (MCMC) (Sorensen and Gianola 2002, Van De Wiel *et al.* 2013). MCMC is a very flexible methodology that can be used to make inference for any Bayesian model, and we can get posterior estimates for any random variable or parameter; marginally, jointly, or functions of them. Setting up a good MCMC algorithm (quick convergence, good mixing, and computationally fast) and evaluating it (convergence and mixing) is challenging for a nonspecialist. Recently, this has improved for animal models as there are now packages available for doing inference for these models with MCMC in R (MCMCglmm; Hadfield 2010) and in BUGS (Lunn *et al.* 2000).

For hierarchical latent Gaussian Markov random field models, a nonsampling-based numerical approximation procedure, the Integrated Nested Laplace Approximation (INLA) has recently been introduced (Rue *et al.* 2009). Using INLA we can calculate marginal posteriors for all parameters and each random effect, as well as the posterior for linear combinations of random effects. INLA is based on direct numerical integration instead of simulations. Rue and Martino (2007) show for several models and datasets that INLA is much faster than MCMC and more accurate for a given computation budget. Faster inference encourages applied researchers to explore more models. Furthermore, this also opens new opportunities to do simulation studies, which for example can be used to explore identifiability issues and to set up tests of specific hypotheses. To perform model selection between GLMMs is not a trivial task (Skrondal and Rabe-Hesketh 2004), and using difference in deviance information criterion (DIC) has been questioned (Fong *et al.* 2010). We suggest using a simulation study to evaluate whether DIC is an appropriate measure for model selection. Fast simulation and inference methods are essential for simulation studies to be computationally feasible. INLA has been used in several fields of statistics, *e.g.*, survival analysis (Martino *et al.* 2011), for spatial GLMM (Eidsvik *et al.* 2009) and in disease mapping (Roos and Held 2011, Schrödle *et al.* 2011). This paper contributes to easier and faster Bayesian inference for both Gaussian and several non-Gaussian animal models by demonstrating that these models fit the INLA-framework and by providing an R-package, AnimalINLA, for doing the inference.

In the section *Materials and Methods*, we introduce the data used in the case studies. Then we briefly revise relevant requirements for using INLA and the possibilities INLA gives, and fully specify the animal models we use. We also present a framework for simulation based testing of the ability of difference in DIC to choose between models with and without genetic effects. Next, results from the synthetic case studies and the house sparrow case studies are presented. Inference is carried out using INLA and for some cases results are compared with MCMC. The article ends with a *Discussion* and *Conclusion*, where the results as well as opportunities and limitations of the INLA framework in quantitative genetics are discussed.

## Materials and Methods

### Data

For the case studies we use data from a natural metapopulation of house sparrow (*Passer domesticus*) on six islands off the coast of Helgeland, Northern Norway (66°N, 13°E). From adults and juveniles (*i.e.*, birds born the same summer) a small blood sample was collected and from adults several morphological traits were measured (including bill depth). The blood samples were used to determine genetic parenthood, and a genetic pedigree for the birds on the study islands could be established. This study system has many qualities for providing data on morphology and fitness-related traits as more than 90% of all birds on the six main study islands were individually ringed. Intensive observation and capture protocols each year gave good estimates of the lifespan of individual birds (a bird was considered dead when it was no longer captured or observed). For a more thorough description of the field work, study area and genetic parenthood analyses, see (Ringsby *et al.* 2002; Jensen *et al.* 2008; Pärn *et al.* 2009) and references therein.

For all case studies we used 1993 to 2002 as our study period, and we used the same pedigree, which consisted of the *n_p_* = 3574 individuals that were present on the six main study islands in this period. The pedigree spanned up to seven generations. For our case studies we used individual data on (1) bill depth, (2) breeding season success, and (3) average reproductive intensity (ARI). For all birds, sex, hatch year, and hatch island were available. The animal model implicitly assumes that missing phenotype observations are missing at random, and hence we (implicitly) have assumed this. However, if the process behind the missing data are connected to the trait of interest, this could result in biased inference of additive genetic variance (Hadfield 2008).

In case study one we considered bill depth. This trait was measured each year (*i.e.*, age) for most individuals over the course of a lifetime. The proportion of individuals that have more than one measurement was 0.4, ranging from two to nine measurements in total. Bill depths were approximately Gaussian distributed (see Supporting Information, Figure S1), and we have measurements for *n_d_* = 1025 birds. Many individuals in the pedigree had missing data for this trait because the bird did not survive until it was 1 year of age. We standardized the data to have mean 0 and variance 1.

In case study two, we considered breeding season success. If at least one of the offspring of an adult bird produced in a given breeding season survived until recruitment, we defined its breeding season a success. A recruit is an offspring that survives up to adult age, *i.e.*, 1 year of age in the house sparrow. Otherwise the breeding season was a failure. The breeding season could be a failure either because the bird did not produce any offspring, or because all its offspring died before recruitment. The data consist of pairs of values (*n_i_*, *y_i_*), where *n_i_* is the number of breeding seasons individual *i* had during the study period (*i.e.*, it was alive and adult) and *y_i_* is the number of successful breeding seasons, *y_i_* ≤ *n_i_*. Individuals that died before their first breeding season (did not recruit) or that emigrated to an island not among the six main study islands have no data. There are *n_d_* = 1182 individuals with data. Of these approximately 71% did not have any successful breeding seasons.

In case study three, we considered data on ARI, *i.e.*, the average number of recruits an individual produced over its lifetime. Data take the form (*n_i_*, *y_i_*) where *n_i_* is identical to *n_i_* in case study two, and *y_i_* is the total number of recruits produced in the study period. For this trait we had data for the same *n_d_* = 1182 individuals as in case study two. *y_i_* ranged from 0 to 10, with mean 0.64. 71% produced no recruits, and about 46% of the 344 individuals that produced one or more recruits produced only one. The datasets are available in File S5.

### Latent Gaussian models and INLA

In this section we give a brief introduction to latent Gaussian models and how INLAs can be used to make approximations for posterior marginals for these models. In general, latent Gaussian models are hierarchical models in which we assume a *n_p_*-dimensional latent field ***x*** to be point-wise observed through *n_d_* ≤ *n_p_* data ***y***, $f\left(y|x\right)=\prod_{i=1}^{n_{d}}f\left(y_{i}|x\right)$. The latent field *x* includes both random and fixed effects and is assumed to have a Gaussian density conditional on hyperparameters ***θ*_1_**: ***x***|***θ*_1_** ∼ N (0, ***Q***^−1^(***θ*_1_**)).

The data ***y*** are assumed to be conditionally independent given the latent field ***x*** and, possibly, some additional hyperparameters *θ*_2_. The model definition is completed by assigning a prior density to the hyperparameters ***θ*** = {***θ***_1_, ***θ***_2_}. In addition, some linear constraints of the form ***Bx*** = *e*, where the matrix ***B*** has rank *k*, may be imposed (Rue *et al.* 2009).

INLA provides a recipe for computing in a fast and accurate way, approximations to marginal posterior densities for the hyperparameters $\tilde{\pi }\left(\theta |y\right)$ and for the latent variables $\tilde{\pi }\left(x_{i}|y\right)$. Such approximations are based on a smart use of Laplace or other related analytical approximations and of numerical integration schemes. As a by-product of the main computations INLA can also compute the DIC (Spiegelhalter *et al.* 2002). DIC is calculated as the expectation of the deviance over the posterior distribution (*E****^x^***^,^***^θ^***(*D*(***θ***, ***x***))) plus the effective number of parameters (*p_D_*), DIC = *E^**x**^*^,^*^***θ***^*(*D*(***θ***, ***x***)) + *p_D_*. For the class of latent Gaussian models INLA is constructed for the deviance can be expressed as $D\left(\theta ,x\right)=-2\sum_{i=1}^{n}\text{log}\;f\left(y_{i}|\theta ,x\right)$, where the sum is over all observations. The posterior mean of the deviance $E^{x,\theta }\left(D\left(\theta ,x\right)\right)=\int_{\theta ,x}D\left(\theta ,x\right)\pi \left(\theta |y\right)\pi \left(x|\theta ,y\right)\text{d}\theta \;\text{d}x$ can therefore be calculated using INLA (Rue *et al.* 2009). *p_D_* is approximated to *n* − *tr*{***Q***(***θ**^m^*)Q*(***θ**^m^*)^−1^}, where *n* is the number of observations and ***θ**^m^* denotes the posterior median of *π*(***θ***|***y***). It is worth to note that another common definition of DIC is to use the posterior mean (instead of median) for the parameters in the deviance. This definition is used in MCMCglmm.

For the INLA methodology to work in a fast and efficient way, latent Gaussian models have to satisfy some additional properties. First, the latent Gaussian model ***x***, often of large dimension, admits conditional independence properties. That is, it is a Gaussian Markov random field (GMRF) with a sparse precision matrix *Q* (Rue and Held 2005). The efficiency of INLA relies, in fact, on efficient algorithms for sparse matrices. Second, because INLA needs to integrate over the hyperparameter space, the dimension of non-Gaussian ***θ*** should not be too large, say ≤ 14, due to the numerical integration scheme and optimization methods used. Finally, each data point *y_i_* depends on the latent Gaussian field only through the linear predictor *η_i_* = *g*(*μ_i_*) where *g*(⋅) is a known link function and *μ_i_* = *E*(*y_i_*|***x***, ***θ***), *i.e.*, *π*(*y_i_*|***x***, ***θ***) = *π*(*y_i_*|*η_i_*, ***θ***). INLA presents several advantages over MCMC based inference: it provides accurate results in just a fraction of the time needed by smart MCMC algorithms, and it does not require convergence diagnostics. Moreover, the R-INLA package (available at www.r-inla.org) makes inference from GRMF models using the INLA methodology easy.

### Animal models

In this section we show that animal models are latent GMRF models, which fits into the INLA framework (see the section *Latent Gaussian models and INLA*). Moreover, we describe in detail the different versions of animal models for which we are interested. Because the focus of this article is to emphasize INLA as a method of inference for animal models, the models in the case studies and synthetic studies are kept simple. For a more in depth introduction to animal models, see (Sorensen and Gianola 2002).

In general, an animal model is a generalized linear mixed model; the observed trait *y_i_*, *i* = 1, …, *n_d_* belongs to an exponential family

$$y_{i}∼\pi \left(y_{i};\mu_{i},\theta_{2}\right),$$

where the expected value *μ_i_* = *E*(*Y_i_*) is linked to a linear predictor *η_i_* through a known link function *g*(⋅), so that *g*(*μ_i_*) = *η_i_*. The linear predictor *η_i_* accounts for the effects of various covariates and the breeding value in an additive way;

$$\eta_{i}=\beta_{0}+z_{i}^{T}\beta +u_{i}+\varepsilon_{i},$$ (1)

where $\beta_{0}$ is an intercept, $\beta =\left(\beta_{1},\ldots ,\beta_{n_{f}}\right)$ are *fixed effects*, *u_i_* individual *i*’s breeding value, *ε_i_* its residual effect, and $z_{i}^{T}$ a known incidence vector. The *fixed effects* (in a frequentist’s framework) account for group-specific effects such as *e.g.*, sex, year of birth, and locality or subpopulation. In a Bayesian framework all parameters are treated as random variables, but out of convenience we refer to ***β***s as fixed effects. The breeding values are genetically linked random effects also known as additive genetic effects. The residual effects are unstructured Gaussian random effects, often named the environmental effect in quantitative genetics. We assign a Gaussian prior to ***β***: $\beta ∼N\left(0,\sigma_{\beta }^{2}I\right)$, where ***I*** is the identity matrix. The residual effects are $\varepsilon ∼N\left(0,\sigma_{e}^{2}I\right)$, where $\sigma_{e}^{2}$ is often referred to as environmental variance.

The breeding values for the population, $u=\left(u_{1},u_{2},\ldots u_{n_{p}}\right)$, are assumed to have a dependency structure corresponding to the pedigree

$$u|A,\sigma_{u}^{2}∼N\left(0,\sigma_{u}^{2}A\right),$$

where ***A*** is the relationship matrix and $\sigma_{u}^{2}$ is the additive genetic variance (see *e.g.*, Lynch and Walsh 1998, Sorensen and Gianola 2002). A GMRF is a multivariate Gaussian model with a conditional independence structure, also known as a Markov structure. The pedigree imposes a Markov structure. If we are interested in individual *i*’s breeding value, and we know its parents, offspring and the other parent(s) of its offspring, other individuals’ breeding value do not give us any extra information. Because of the fact that the breeding values forms a GMRF, the inverse of the relationship matrix, ***A*^−1^**, is a sparse matrix (Steinsland and Jensen 2010). ***A*^−1^** can be calculated from the pedigree (Quaas 1976).

Note that there might be more individuals in the pedigree than individuals with observations, *n_d_* ≤ *n_p_*, and we have assumed an indexing such that *u_i_* corresponds to *y_i_*. The hyperparameters $\left(\sigma_{u}^{2},\sigma_{e}^{2}\right)$ are assigned inverse gamma priors. Furthermore, to avoid identification problems we include a common intercept and constrain the levels of each factors to sum to zero (see Steinsland and Jensen 2010).

The animal model as described previously is a latent GMRF model where the latent field is ***x*** = (***β***, ***u***) and the hyperparameter vector *θ* includes the variances $\left(\sigma_{u}^{2},\sigma_{e}^{2}\right)$ and, possibly, the parameters in the likelihood function. The precision matrix for the latent field *x* is sparse because the inverse of ***A*** is sparse. Moreover, the likelihood of each data point depends on the latent field only through the linear predictor *η_i_* defined in equation 1. Therefore, INLA can be applied to the animal model.

In our analyses we might be interested in marginal posterior for individual breeding values, *u_i_*, fixed effects ***β***, the additive genetic variance $\sigma_{u}^{2}$, the residual variance $\sigma_{e}^{2}$, the heritability *h*^2^, or to evaluate the model using DIC. The heritability is loosely speaking the proportion of the variability the genes account for in a phenotypic trait. Precise definitions of heritability are given in subsequent subsections. In addition, it might be interesting to look at linear combinations of breeding values $\sum_{i\in C}w_{i}u_{i}$, where *w_i_* are weights, for example the mean of breeding values for different cohorts.

#### Animal model for Gaussian data:

For many continuous traits, such as the bill depth of house sparrows, it is natural to assume a Gaussian likelihood with an identity link function, *η_i_* = *μ_i_*. The animal model can then be written as: $y_{i}∼N\left(\mu_{i},\sigma_{e}^{2}\right)$, where the linear predictor is modeled as in (1) and the variance $\sigma_{e}^{2}$ is the variance of the residual effects. The model can be formulated in the INLA framework with likelihood $y_{i}|\eta_{i}∼N\left(\eta_{i},\sigma_{e}^{2}\right)$ and latent field $\eta_{i}=\beta_{0}+z_{i}^{T}\beta +u_{i}$.

In many datasets there are repeated measurements for some individuals. A common modeling approach is then to include an individual specific random effect *ind_i_* for each individual. Let *y_ij_* denote measurement *j* of individual *i*. The likelihood is $y_{ij}|\eta_{i}∼N\left(\eta_{i},\sigma_{e}^{2}\right)$, and the latent field $\eta_{i}=\beta_{0}+z_{i}^{T}\beta +u_{i}+ind_{i}$. This redefines the variance interpretation, and we can interpret $\sigma_{\mathrm{ind}}^{2}$ as a special environmental variance (variation of the individuals’ trait values through life), and $\sigma_{e}^{2}$ as the measurement error or unexplained (environmental) variance (Lynch and Walsh 1998).

In general, and in the Gaussian case, the narrow sense heritability is defined as the proportion of the phenotypic variance that is caused by additive genetic variance (Lynch and Walsh 1998)

$$h^{2}=\frac{\sigma_{u}^{2}}{\sigma_{u}^{2}+\sigma_{e}^{2}}.$$ (2)

Although it is easy to use the INLA methodology to compute posterior marginals of hyperparameters, posteriors for functions of more than one hyperparameter, *e.g.*, *h*^2^, become computationally inconvenient. This can be solved by reformulating the model in the INLA framework (see File S1 for this model formulation, and how to use it).

#### Animal model for binomial data:

With binomial data, the animal model is defined as: *y_i_* ~ Bin(*n_i_*, *p_i_*) *i* = 1, …, *n_d_*, where *n_i_* is the numer of trials and *p_i_* is the probability of success. Moreover, we assume a logit link function, so that the linear predictor is defined as: $\eta_{i}=\text{logit}\left(p_{i}\right)=\text{log}\left(\frac{p_{i}}{1-p_{i}}\right)$. The linear predictor is then modeled as in (1). In the binary case (*n_i_* = 1) the variance of the non-structured random effect $\sigma_{e}^{2}$ is confounded with the link, and is not identifiable (Sorensen and Gianola 2002) because the individual effects are already accounted for through the link and the likelihood. Therefore, we omit *ε* from the linear predictor and use this linear predictor for all binomial models

$$\eta_{i}=\beta_{0}+z_{i}^{T}\beta +u_{i}.$$ (3)

For binomial data, it is not immediately obvious how to define the heritability of the trait. The most common definition is derived from the idea that there exists a latent (unobserved) continuous trait called liability *l_i_* such that we register a success if *l_i_* < 0 and a failure if *l_i_* > 0 (Dempster and Lerner 1950). The definition of heritability depends also on the type of the link function and in the case of the logistic function it is

$$h^{2}=\frac{\sigma_{u}^{2}}{\sigma_{u}^{2}+\frac{\pi^{2}}{3}}$$ (4)

were $\pi^{2}/3$ is the variance of a logistic variable (see Vazquez *et al.* 2009). Note that the heritability on the latent scale does not correspond to the proportion of explained variance in the phenotype, *e.g.*, the binomial data. For a discussion on heritability for non-Gaussian traits, see (Dempster and Lerner 1950, Visscher *et al.* 2008). The binomial animal model is a latent Gaussian model with only one non-Gaussian hyperparameter, $\theta =\sigma_{u}^{2}$. The heritability, as defined in (4), is a function of only one random variable, $\sigma_{u}^{2}$, and can therefore easily be calculated from $\sigma_{u}^{2}$’s marginal posterior distribution.

#### Animal model for (zero-inflated) Poisson data:

Count data are often modeled as Poisson distributed: *y_i_* ~ Poisson (*μ_i_*) with *μ_i_* = *E_i_λ_i_*, where *E_i_* is the known exposure, *e.g.*, the lifetime, and *λ_i_* is the intensity, *e.g.*, the annual reproductive success. We assume an exponential link function *η_i_* = log(*λ_i_*), and model the linear predictor ***η*** as in (3).

Datasets which are almost Poisson, but have too many zero-observations, often occur. Then a zero-inflated Poisson (ZIP) distribution might be useful. ZIP models are a mixture of a Poisson distribution and a distribution with point mass one at zero. There are several versions of zero-inflated Poisson, we will use *ZIP*(*p*, *μ_i_*) defined as: Prob(*y*|…) = *p* × 1_[_*_y_*_=0]_ + (1 − *p*) × Poisson(*y*; *μ_i_*), where 1_[_*_y_*_= 0]_ is an indicator function and Poisson(*y*; *μ_i_*) indicates the Poisson likelihood with mean *μ_i_*, and *p* is the proportion of extra zeros. Poisson and zero-inflated Poisson animal models are latent Gaussian fields with hyperparameter vectors $\theta =\sigma_{u}^{2}$ and $\theta =\left(\sigma_{u}^{2},p\right)$, respectively.

In the Poisson case it has been proposed that the heritability on the log scale can be defined as (Foulley *et al.* 1987, Matos *et al.* 1997, Vazquez *et al.* 2009)

$$h^{2}=\frac{\sigma_{u}^{2}}{\sigma_{u}^{2}+\lambda^{-1}}$$ (5)

where *λ* is the average intensity; $\lambda =\frac{1}{n_{d}}\sum_{i=1}^{n_{d}}\lambda_{i}=\frac{1}{n_{d}}\sum_{i=1}^{n_{d}}\text{exp}\left(\eta_{i}\right)$.

The heritability (5) is then a function of one hyper-parameter and the random variable *λ* which is a linear combination of functions of predictors *η_i_*. Such a quantity is (at least currently) not possible to compute using R-INLA. An approximated estimate of *h*^2^ can be computed by using a point estimate for *λ* together with the marginal posterior of $\sigma_{u}^{2}$. The point estimate can either be calculated directly from data, or by plugging in point estimates for the predictors ***η***. With this model we calculate the heritability of the intensity, *e.g.*, ARI, although the data are individual lifetime reproductive success. If the heritability of lifetime reproductive success (LRS) is of interest, this can be estimated by setting the exposure *E_i_* = 1 (and only using individuals that are uncensored at either end of the study period).

#### Simulation-based evaluation of DIC:

We often want to check whether an additive genetic effect should be included in the model or not. In a classical approach to hypothesis testing, this corresponds to a null hypothesis of no genetic effects (no heritability), and an alternative hypothesis of some genetic effect (heritability). This can be seen as a model selection between a model without genetic effects and an animal model. That is, in our formulation from the section *Animal Models*, between a model with some likelihood and latent field,

$$H_{0}:\eta_{i}=\beta_{0}+z_{i}^{T}\beta$$ (6)

or

$$H_{1}:\eta_{i}=\beta_{0}+z_{i}^{T}\beta +u_{i}.$$ (7)

To evaluate the ability of the difference in DIC to correctly choose between these models we suggest the use of simulations. We use the pedigree, explanatory variables, priors, and missing structure of the dataset and a model we want to compare. To find an estimate of the probability of type I error, concluding that there are genetic effects when in truth there is none, we sample *S* new data sets from a model without genetic effects, *i.e.*, with (6), and impose the same missing structure as in the original data set. For each of these *S* data sets we fit both models, *i.e.*, with and without genetic effects, and find the difference in DIC, ΔDIC = DIC model (6) − DIC model (7). The resulting *S* values of ΔDIC is an approximation to the sampling distribution of ΔDIC under the null hypothesis. If we use the recommended limit of ΔDIC > 10 to reject the null-hypothesis, we can find an estimate of the corresponding significance level from the proportion of the *S* ΔDIC values larger than 10. We can also chose a significance level *α*, and find the corresponding limit of ΔDIC from the simulations.

The other important quantity regarding hypothesis tests is the power function of the test, *i.e.*, the probability the *H*_0_ is rejected when there are some genetic effects. To estimate the power for a specific value of $\sigma_{u}^{2}$ or *h*^2^, we follow the same simulation approach, except that simulations are now done from a model with genetic effects, *i.e.*, from (7) with $\sigma_{u}^{2}$ as chosen. The proportion of these *S* Δ*DIC* values larger than our chosen limit is an estimate of the power for a given $\sigma_{u}^{2}$.

## Results

### Synthetic case studies

In this section we illustrate the INLA methodology using a series of synthetic case studies for the models (described in the section *Animal models*). We report here results for the Gaussian and the Binomial model. For corresponding results for the Poisson model see Table S1.

To make our simulated data set as realistic as possible we do the following: first, we simulate data based on the pedigree of the house sparrow dataset with *n_p_* = 3574 individuals (as described in the section *Data*). Second, we replicate in the simulated data set the same missing data structure that we find in the house sparrow data set. Inference is done using the AnimalINLA package. See File S3 for R code. As priors for $\sigma_{u}^{2}$, $\sigma_{e}^{2}$, and $\sigma_{\beta }^{2}$ we use inverse gamma distribution InvGamma(*a*, *b*) (parametrized such that it has mean $\left(\frac{b}{a-1}\right)$ and variance $\left(\frac{b^{2}}{{\left(a-1\right)}^{2}\left(a-2\right)}\right)$). InvGamma(0.5, 0.5) for $\sigma_{u}^{2}$ and $\sigma_{e}^{2}$ and InvGamma(*e*, *e*^−10^) for $\sigma_{\beta }^{2}$.

#### Synthetic Gaussian case study:

In our first experiment we simulated Gaussian data from:

$$y_{i}|\mu_{i},\sigma_{e}^{2}∼N\left(\mu_{i},\sigma_{e}^{2}\right)$$ (8)

$$\eta_{i}=\mu_{i}=\beta_{0}+u_{i}$$ (9)

where $u|A,\sigma_{u}^{2}∼N\left(0,\sigma_{u}^{2}A\right)$, and ***A*^−1^** is computed from the house sparrow pedigree.

We simulate data for *β*_0_ = 0 and values of $\sigma_{u}^{2}$ and $\sigma_{e}^{2}$ between 0 and 1 such that $\sigma_{u}^{2}+\sigma_{e}^{2}=1$. Moreover, we assume as missing all measurements that are missing for bill depth in the house sparrow data set. We follow Steinsland and Jensen (2010) and fit the model assuming a sum to zero constraint on the breeding values, $\sum u_{i}=0$. In this experiment we are interested in the variance parameters and use the model formulation described in the section *Animal model for Gaussian data* (*i.e.*, MF1 in File S1).

**Figure 1:**
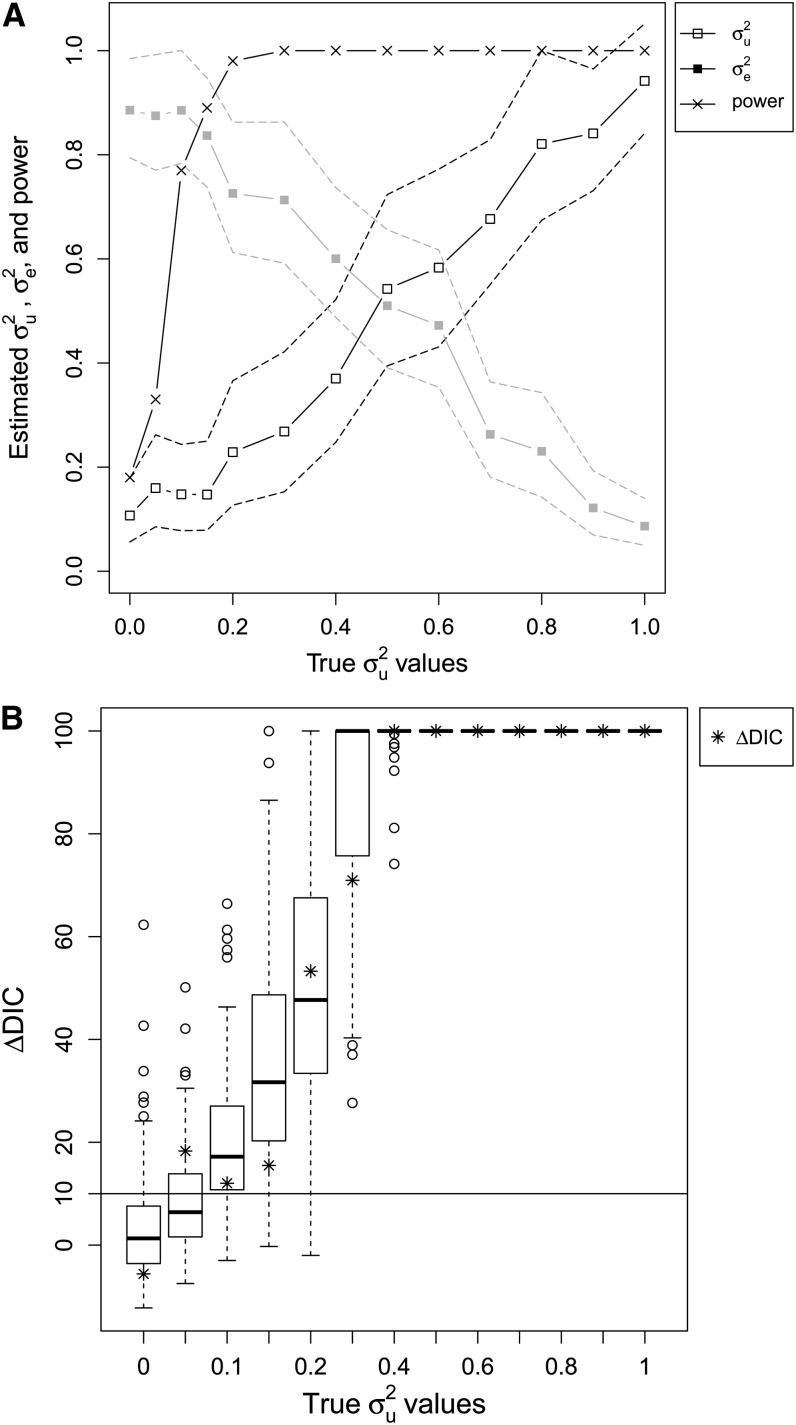
Results from the synthetic Gaussian case study. (A) Posterior mean (solid lines) with 95% CI (dashed lines) for $\sigma_{u}^{2}$ (black and open squares) and $\sigma_{e}^{2}$ (gray and closed squares) from the Gaussian synthetic case study against the value of $\sigma_{u}^{2}$ used in the simulations (together with $\sigma_{e}^{2}=1-\sigma_{u}^{2}$). The power of a model selection test using ΔDIC > 10 as limit is plotted as x and solid lines. The power is estimated using the simulation approach described in the text. (B) Boxplots of simulated values of ΔDIC against the value of $\sigma_{u}^{2}$ used in the simulations (together with $\sigma_{e}^{2}=1-\sigma_{u}^{2}$). The values of ΔDIC from the synthetic case study are plotted as stars. ΔDIC equal to 10 is indicated by a horizontal line.

Figure 1A shows the estimated posterior mean together with standard deviations, and the 95% credible interval (CI) for $\sigma_{u}^{2}$ and $\sigma_{e}^{2}$. The posterior means are quite close to the true values of $\sigma_{u}^{2}$ and $\sigma_{e}^{2}$, with small standard deviations and 95% CIs that contain the true value. However, for small values for $\sigma_{u}^{2}$ (<0.1) the posterior mean of the genetic variance seems to be systematically different from the parameter value used in the simulations. We will refer to this as a systematic error. It is caused by influential priors (discussed in the house sparrow case studies). As the likelihood is Gaussian, the Laplace approximation is exact and hence its accuracy comes from the numerical integration scheme.

For each simulated data set we also fit a model without genetic effect, hence where the model in (8) and (9) simplifies to:

$$y_{i}|\mu_{i},\sigma_{e}^{2}∼N\left(\mu_{i},\sigma_{e}^{2}\right)$$ (10)

$$\eta_{i}=\mu_{i}=\beta_{0}.$$ (11)

We now test, using ΔDIC whether a model with or without genetic affects will be chosen. The ΔDIC results are presented as stars (∗) in Figure 1B, and we find that using a limit ΔDIC > 10 we chose the animal model for $\sigma_{u}^{2}\geq 0.05$.

To evaluate the ability of ΔDIC to choose between models, we use the simulation methodology suggested in the section *Simulation-based evaluation of DIC*. We use the same sets of $\left(\sigma_{u}^{2},\sigma_{e}^{2}\right)$ as in the synthetic case study and simulated *S* = 100 synthetic data sets for each parameter sets. Boxplots of the corresponding ΔDIC are found in Figure 1B, whereas the power of the test for the different sets of $\left(\sigma_{u}^{2},\sigma_{e}^{2}\right)$ are given in Figure 1A. We first notice that the significance level (the power for $\sigma_{u}^{2}=0$) is approximately 0.18. The power rapidly increases, and for $\sigma_{u}^{2}=0.1$ it is 0.77, and already for $\sigma_{u}^{2}=0.2$ it is 0.98. Hence, for Gaussian data with pedigree and missing structure as the house sparrow case study using difference in DIC as model selection criteria we have a good chance of identifying additive genetic variance above 0.1.

#### Synthetic binomial case study:

Binomial data can be challenging to analyze, especially when the number of trials *n_i_* is very low (Fong *et al.* 2010). To analyze the performance of INLA for binomial data we have carried out different simulation studies and compared the estimates obtained with INLA with those obtained using MCMC (MCMCglmm; Hadfield 2010). We simulate data from the model *y_i_*|*p_i_* ~ Bin(*n_i_*, *p_i_*) with a logit link function *η_i_* = logit(*p_i_*) = α + *u_i_*. Where $u|A,\sigma_{u}^{2}∼N\left(0,\sigma_{u}^{2}A\right)$, and ***A*^−1^** is computed from the house sparrow pedigree. We simulate data for *α* = 0 and values of $\sigma_{u}^{2}$ such that the corresponding heritability, computed as in equation (4), varies between 0 and 1.

**Figure 2:**
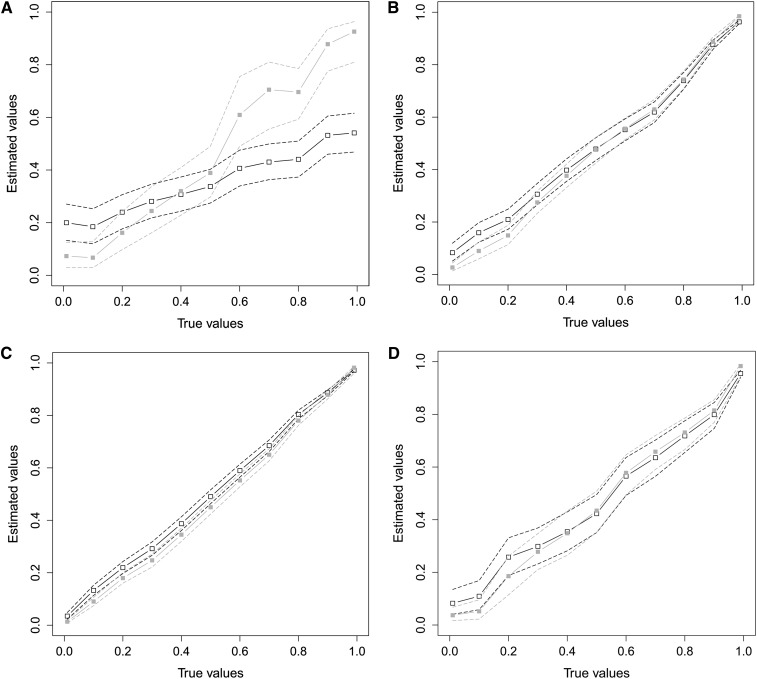
Results from the synthetic Binomial case study. True *vs.* estimated heritability: posterior mean (solid line) and 95% credible intervals (dashed lines) for INLA (black, open squares) and MCMC (gray, closed squares). The number of trials is in (A) 1, (B) 2, (C) uniform between 1 and 9, and (D) distributed as in the house sparrow data set.

In our first experiment we let *n_i_* = 1, ∀*i* = 1, …, *n_p_*, hence we have binary data for all the individuals in the pedigree. This case is, in general, particularly difficult, because with no replicates for any of the individuals the genetic variance is not identifiable (Sorensen and Gianola 2002). When we look at the posterior estimate for $\sigma_{u}^{2}$, we see that the performance of INLA is quite bad (see Figure 2A). Looking at Figure 2A, we find that the posterior of the heritability differs between MCMC and INLA, MCMC follows the true value, INLA does not. INLA is based on a Gaussian approximation of the log-likelihood functions which, in this case, has a very non-Gaussian distribution, and the Laplace approximation is poor. Although MCMC follows the true value is has quite large credible intervals. The dependency structure induced by the house sparrow pedigree is not strong enough to allow for a precise estimation of the genetic variance. In practice, it is almost impossible to distinguish between cases with high and low heritability of the binary trait. We further find that for small heritability the estimates have systematic errors, as in the Gaussian case.

The performance of INLA improves very quickly with increasing number of trials. In our second experiment we let *n_i_* = 2, ∀*i* = 1, …, *n_p_*, hence we have two trials for each individual in the pedigree. In this case, the presence of replicated measures makes it possible to estimate the genetic variance more accurately. Figure 2B shows that the posterior means computed by INLA are very close to those computed using MCMC and close to the true value of *h*^2^. We still see a small systematic error for small values of *h*^2^ but not such that it would be problematic in a real data scenario. Even better estimates are obtained in the third experiment where the number of trials *n_i_* changes from individual to individual in the pedigree and is randomly sampled between 1 and 9 (see Figure 2C).

In the last experiment the number of trials *n_i_* is as in the house sparrow breeding season success data set (see the section *Data*). Moreover, in the simulated data we reproduce the same missing structure as in the real data set. In this experiment the number of trials is sampled uniformly between 1 and 9, and there are 1182 individuals with data. That is, for more than 65% of the individuals in the pedigree the trait under consideration was not recorded. Results shown in Figure 2D, are similar to those for the two previous cases. The estimates seem to be rather accurate with a small systematic error for very small values of the heritability. Moreover, results from INLA agree well with those from MCMC. In this experiment we have larger CI around the posterior mean when compared to the one in Figure 2C. This is attributable to the presence of missing data.

To test prior sensitivity, we performed a sensitivity analysis for all three likelihoods and for both no heritability (*h*^2^ = 0), and high heritabilities. Each dataset was analyzed with five different priors for $\sigma_{u}^{2}$ and, when relevant, $\sigma_{e}^{2}$ ; InvGamma(*a*, *b*) with a = b = 0.0001, 0.01, 0.5, 1, 10. The sensitivity analyses are described in File S2 and results are shown in Figure S3. The general findings are that for low heritabilities the results are very prior sensitive, while for higher heritabilities only extremely informative priors (*a* = *b* = 10) change the posterior considerably.

### House sparrow case studies

In this section we analyze the data introduced in the section *Data* using the animal models in the section *Animal models*. To perform inference we use INLA, described in the section *Latent Gaussian models and INLA*. We have three case studies; bill depth, breeding season success, and ARI. For each case study, we first do model comparison using DIC to choose which fixed effects (sex, hatch year, and hatch island) and random effect (additive genetic effect) to include in our model. For the best model we do further analysis according to the chosen model and the case study. This includes estimating parameters, heritability and mean breeding values for each cohort.

For all models we use these priors: $\beta ∼N\left(0,\sigma_{\beta }^{2}=2.2\cdot 10^{4}\right)$, $\sigma_{u}^{2}∼\text{InvGamma}\left(0.5,0.5\right)$ and (when needed) $\sigma_{e}^{2}∼\text{InvGamma}\left(0.5,0.5\right)$. To choose the best model, we start with the full model and remove one variable at the time in a stepwise manner. In each step all nested models are examined, but only the one with lowest DIC (*i.e.*, the best one at each step) is reported in Table 1.

**Table 1:** Model selection in house sparrow case studies

	DIC	Best Model
Bill depth~		
Year + sex + island + age + u	2468.208	
Year + sex + island + u	2466.415	*
Year + sex + u	2467.077	
Year + u	2477.196	
Year	2591.390	
Breeding season success~		
Sex + year + island + u	1718.687	
Sex + year + island	1709.878	*
Year + island	1710.776	
Year	1713.180	
ARI~		
Year + sex + island + u	2275.140	*
Year + sex + island	2275.729	
Year + sex	2283.010	
Sex	2291.700	

Deviance information criteria (DIC) for different models explaining variance in bill depth, breeding season success, and average reproductive intensity (ARI) of Norwegian house sparrows are shown. Best models (lowest DIC) for bill depth, breeding season success and ARI are indicated by *.

### Bill depth

Bill depth is a Gaussian trait, and we use the animal model described in the section *Animal model for Gaussian data*. We first looked at bill depth when first (time) measured and age of the individual at that time. The full model can be written as; *η_i_* = *β*_0_ + *β*_sex(_*_i_*_)_ + *β*_year(_*_i_*_)_ + *β*_island(_*_i_*_)_ + *β*_age_*x*_age_(*i*) + *u_i_*, where *sex*, *year*, and *island* are group-level factors and *x*_age_(*i*) is a linear covariate with same prior as for ***β***. The results from the model selection procedure are presented in Table 1. We see that the full model without age as a linear effect turns out to be the best;

$$\eta_{i}=\beta_{0}+\beta_{\text{sex}\left(i\right)}+\beta_{\text{year}\left(i\right)}+\beta_{\text{island}\left(i\right)}+u_{i}.$$ (12)

Our further analyses for bill depth are based on this model.

**Figure 3:**
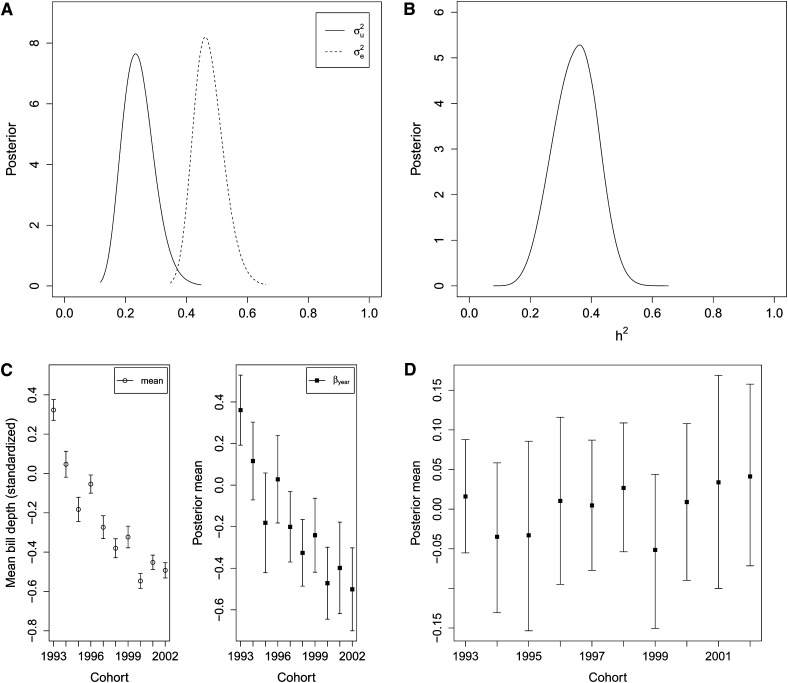
Results from house sparrow case study on bill depth. All posterior estimates are obtained using INLA. (A) Posterior distribution of $\sigma_{u}^{2}$ (solid line) and $\sigma_{e}^{2}$ (dotted line). (B) Posterior distribution of heritabililty *h*^2^. (C) Left: Mean phenotopic (standardized) bill depth for each hatch year (with 95% confidence interval). (C) Right: Posterior mean (with 95% credible interval) of the levels *β_year_* for the factor *year*. (D) Posterior mean (with 95% credible interval) for average breeding values for each hatch year.

We find the marginal posterior distribution for the variances; $\sigma_{u}^{2}$ has posterior mean 0.24 (SD 0.05) and 95% CI 0.15−0.35. For $\sigma_{e}^{2}$ we get a posterior mean 0.47 (SD 0.05) with 95% CI 0.39−0.58. We also calculate the marginal posterior of *h*^2^ using MF2 (see File S1); mean 0.35 (SD = 0.07) with 95% CI 0.21−0.48. The posteriors for $\sigma_{u}^{2}$, $\sigma_{e}^{2}$, and *h*^2^ are plotted in Figure 3, A and B.

To explore interesting features and evolutionary processes that may influence our study system we investigate trends in the breeding values over years (Sorensen *et al.* 1994), by estimating linear combinations of breeding values. We find the posterior distribution of average breeding values for each hatch year *year* (*i.e.*, cohort); $a_{year}=\frac{1}{n_{year}}\sum_{i\in C_{year}}u_{i}$, where *n_year_* is the number of individuals with hatch year *year*, and the sum is over all these individuals. We fit the model specified in equation 12 (which includes hatch year as a factor), and the results are given in Figure 3C (posterior of *β_year_*) and Figure 3D (posteriors of mean breeding values for each cohort). We also calculate the posterior of the difference in average breeding values between the first (1993) and last (2002) cohorts in the study; *a_diff_* = *a*_1993_ − *a*_2002_, which gave a posterior mean −0.025 (SD 0.068) and 95% CI −0.161 – 0.108. The posterior marginal of the difference is given in Figure S5. From Figure 3, C and D we see that almost all the differences in the phenotype seems to be explained by the year specific fixed factor *β_year_*, and from Figure 3D there seems to be no trends in the breeding values, and hence no microevolution is going on. This is further supported by the posterior of the difference in breeding values between 1993 and 2002 cohorts *a*_diff_|*y*, where 0 is well within a 95% credible interval.

We also model an individual specific random effect *ind_i_*, to include repeated measurement *j* for individual *i*, with latent field *η_i_* = *β*_0_ + *β*_sex(_*_i_*_)_ + *β*_year(_*_i_*_)_ + *β*_island(_*_i_*_)_ + *u_i_* + *ind_i_*, where *ind_i_* is a independent random effect and $\sigma_{\mathrm{ind}}^{2}∼\text{InvGamma}\left(1,0.001\right)$. When including individual as a random effect, we find that the marginal posterior distribution for the variances; $\sigma_{u}^{2}$ has posterior mean 0.25 (SD .05) and 95% CI 0.17−0.37. For $\sigma_{e}^{2}$ we get a posterior mean 0.42 (SD 0.02) with 95% CI 0.38−0.46. For $\sigma_{\mathrm{ind}}^{2}$ we get the posterior mean 0.13 (SD 0.04) and 95% CI 0.07−0.21.

### Breeding season success

Breeding season success is the number of breeding seasons that is a success, *i.e.*, results in at least one recruit. These data are in nature binomial, and are analyzed using the animal model in the section *Animal model for Binomial data*. We start with the full model:

$$\eta_{i}=\beta_{0}+\beta_{\text{sex}\left(i\right)}+\beta_{\text{year}\left(i\right)}+\beta_{\text{island}\left(i\right)}+u_{i},$$ (13)

where the elements are described under equation 12. Results from the model selection procedure are reported in Table 1. We find that the best model does not include linear additive genetic effects, and hence that the inherited part of breeding season success probably is very close to zero.

However, if we use the full model (equation 13) to estimate $\sigma_{u}^{2}$ we get posterior mean 0.13, SD 0.05, and 95% CI 0.07−0.24. Furthermore, using (4) gives posterior heritability with mean 0.04 (SD 0.01) and 95% CI 0.02−0.07. These estimates are similar to those from the synthetic dataset when heritability is equal or close to zero in the section *Synthetic binomial case study* (Figure 2).

### Average reproductive intensity

**Figure 4:**
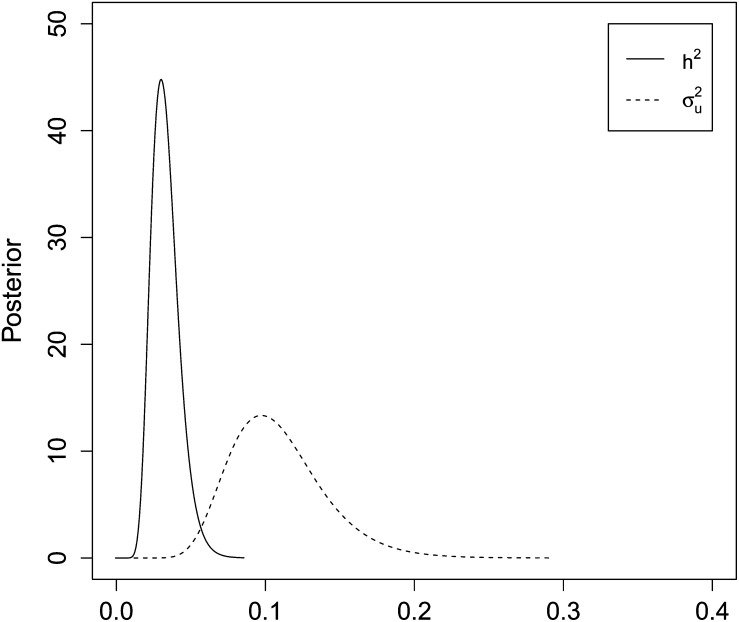
Results from house sparrow case study on ARI. Posterior distribution of the heritability (*h*^2^) and additive genetic variance $\left(\sigma_{u}^{2}\right)$ of a zero-inflated Poisson distributed trait, average reproductive success, in Norwegian house sparrows.

ARI for an individual is the average number of recruits it produces during its lifetime. Thus, we are modeling data on LRS in such a way that lifetime is controlled for and the likelihood of any estimate of heritability will be for the ARI. This is count data, and we analyzed this trait by using the animal model in the section *Animal model for (zero-inflated) Poisson data* with *E_i_* = *n_i_*, where *n_i_* is the number of breeding seasons individual *i* was alive during the study period. Because of the large number of zeros, we suspected that we needed a model that accounts for overdispersion. Therefore, we first fitted the full model as in equation 13 with two different likelihood models; Poisson (DIC = 2421.465) and zero-inflated Poisson (DIC = 2275.140). Because zero-inflated Poisson gave lowest DIC, we proceeded with this likelihood when choosing which fixed and random effects to include in the model. Also the histogram of LRS divided by lifespan indicated a zero-inflated Poisson distribution (see Figure S6). The model with lowest DIC is the full model (equation 13), although very close in DIC to the model without additive genetic effects (Table 1). We proceeded with the full model in our analysis of ARI. Hence, the results suggest that annual reproductive intensity might be heritable. Accordingly, the posterior for $\sigma_{u}^{2}$ is 0.11 (SD 0.03) with 95% CI 0.06−0.18. To obtain the posterior of *h*^2^ defined as in (5), we plugged in the point estimate $\lambda^{*}=\frac{\sum y_{i}}{\sum n_{i}}$. This gives a posterior mean of the heritability of 0.03 (SD 0.01), 95% CI 0.02−0.05. Posterior distributions for $\sigma_{u}^{2}$ and *h*^2^ are given in Figure 4.

The models used in the case studies can be extended. For example when modeling breeding season success, we might want year as a specific explanatory variable. This can be done by modeling it as repeated binary trait (each breeding season). When one uses binomial and Poisson likelihoods overdispersion is often a challenge. Common solutions are to include a random effect to the latent field or to use beta-binomial and negative binomial likelihoods, respectively, instead. All these options are available in INLA and R-codes for how to implement a random effect for Gaussian, binomal and Poisson likelihoods are presented in File S4. For small values of $\sigma_{u}^{2}$ we observed systematic errors and prior sensitivity in all case studies. Priors for variances are discussed in Gelman (2006), and this topic should be further investigated, but it is outside the scope of this work.

### Computation time

To compare the computation time used by INLA and MCMC, inference for the Gaussian case study and for the synthetic Gaussian case study for a large pedigree was performed with both MCMCglmm and INLA. All computation times reported are for a dual-core 2.67-GHz laptop. We visually inspect the posteriors of $\sigma_{u}^{2}$ and $\sigma_{e}^{2}$ of INLA and MCMCglmm for an increasing number of iterations ((10.000, 100.000, 200.000) for the Gaussian case study and (10.000, 100.000, 500.000) for the synthetic Gaussian case study). MCMCglmm gave the same estimates as INLA (see Figure S2 and Figure S4). For the Gaussian case study, the computation time for INLA was 7 sec for both model formulations for Gaussian data. For 200,000 iterations MCMCglmm used 17 min to achieve about the same accuracy as INLA (the Monte Carlo error is, however, still visible).

To demonstrate the fast inference of INLA, we created a large pedigree from the existing house sparrow pedigree by merging 28 identical pedigrees and simulated data based on this pedigree with *n_p_* = 100,072 individuals. We simulated Gaussian data for *β*_0_ = 0 and for $\sigma_{u}^{2}=0.4$ and $\sigma_{e}^{2}=0.6$ as in section *Synthetic Gaussian case study*, with data for all individuals as in the pedigree. The computation time for INLA was 7.4 min. To achieve approximately the same accuracy as INLA, 500,000 iterations in MCMCglmm were needed; this took 17.9 hr, which is 145 times longer than INLA. As shown in Figure S4, we found that even for 500,000 iterations, the Monte Carlo error was still visible.

## Discussion

We compared inference obtained for animal models by using INLA and MCMC. The general conclusion is that INLA is a fast and accurate approximation method that gives us the opportunity to perform simulation studies to explore models and identifiability issues. However, INLA is less flexible than MCMC methods, and we experienced this in case study three (ARI-data, Poisson likelihood), for which we were not able to calculate the heritability as defined in (5) using INLA. Although an approximated estimate could be obtained, in the Gaussian case heritability estimates can be obtained with INLA by using a tailored reparametrization. In the synthetic case study of binary traits, we experienced that INLA performed poorly for the additive genetic variance $\sigma_{u}^{2}$ (for the pedigree we have used). Hence, we recommend that one should not use INLA for a binary animal model unless a simulation study suggests that INLA gives correct results for the pedigree and missing data structure of the case in question.

Our study of average breeding value for bill depth (Figure 3D) did not indicate any change across cohorts. The posteriors of the levels of the factor *year*; (*β_year_*, Figure 3C) suggests that the observed phenotypic change is influenced by changes in the environment. Note that the estimates of linear combinations we obtain here take into account dependencies and uncertainties of breeding values and parameters. Hence, they do not suffer from the same systematic errors as when using regression on BLUP estimates obtained from REML-based analyses as discussed in Wilson *et al.* (2009) and Hadfield *et al.* (2010).

When we extend the animal model for bill depth to account for repeated measurements and use individual as a random effect, we find that $\sigma_{e}^{2}$ decreases. Some of the variance can now be explained by the individual effects, and the residual error can be interpreted as a measurement error and/or individual changes in bill depth during a house sparrow's lifetime. The additive genetic variance did however remain the same.

The evolutionary process of selection may act on multiple (genetically linked) traits simultaneously (Lande and Arnold 1983). Hence, genetic correlations may impose constraints on the evolution of a given trait. A multivariate animal model which incorporates multiple traits simultaneously to estimate the genetic correlation between traits (*i.e.*, the additive genetic variance-covariance matrix) is therefore often of interest (Meyer 1991; Lynch and Walsh 1998; Kruuk 2004; Jensen *et al.* 2008). This extension of the animal model is in principle possible using the INLA methodology, but is limited by the number of hyperparameters and is not yet implemented in the software.

A growing interest in quantitative genetics is for instance the use of genome-based data, such as high-density single-nucleotide polymorphism, in combination with a pedigree to be able to do better predictions of phenotypes (*e.g.*, Goddard and Hayes 2009). The model by Yang and Tempelman (2012) uses pedigree information together with a single-nucleotide polymorphism model that takes into account the within chromosome dependency. With some adjustments it will fit the INLA framework, but this requires further research.

Both breeding season success and annual reproductive success are traits closely related to fitness. Fitness related traits have previously been found to be largely influenced by the environment and thus have low heritability (Merilä and Sheldon 2000). In our study the breeding season success was not found to be heritable, and annual reproductive success had very low heritability. Consequently, our results coincide with other studies in natural populations (see Jones 1987; Merilä and Sheldon 2000), finding low heritability for fitness-related traits. Note however that the environmental variance of fitness related traits usually is large, and that this will result in a low heritability of such traits even if they have some additive genetic variance (*e.g.*, Foerster *et al.* 2007). Consequently, despite their low heritability these traits may still have evolutionary potential (Hansen *et al.* 2011).

In this work we demonstrated that INLA provides a suitable methodology for performing inference for a range of animal models. We have showed that because of the fast and accurate inference of INLA, we are able to use simulation studies for large pedigrees to examine different models and demonstrated the applicability of examining the difference in DIC as a method for model selection when using simulation studies. In our case studies we considered models with additive genetic effects (breeding values ***u***), individual effects (environmental effects ***ε***), and several fixed effects. These case studies required animal models with Gaussian, Binomial (with logit link), Poisson, and zero-inflated Poisson (with log link) likelihoods. Animal models might have a range of likelihoods. The R-INLA software also support different zero-inflated Gaussian and Binomial likelihoods, survival models (exponential, Weibull, and Cox likelihoods), Student’s *t*, and skew-normal likelihoods (see www.r-inla.org). It is also straightforward to make inference with INLA for animal models extended with other additive random effects, such as maternal effects or litter effects, as well as covariates.

The R-package AnimalINLA has been developed for performing inference using INLA for animal models with likelihoods applied in this paper. It can be downloaded at www.r-inla.org. This package includes functionality for calculating the inverse of the relationship matrix ***A*** from a pedigree and simulation of data from pedigree. Furthermore, there are tailored functions for finding posteriors for $\sigma_{u}^{2}$, $\sigma_{e}^{2}$, the heritability for Gaussian, binomial and Poisson likelihoods and linear combinations such as $\sum_{i\in C}u_{i}$. These functions use R-INLA with suitable default settings. The R-INLA code is also included to give a good starting point to users who wants to make modifications, *e.g.*, other likelihoods or more random effects. Through providing easy to use software which gives results fast we hope Bayesian animal models become accessible to a wider audience of biologists and animal breeders.

## Supplementary Material

Supporting Information

## References

[bib1] Bolker B. Brooks M. Clark C. J. Geange S. W. Poulsen J. R., 2009 Generalized linear mixed models: a practical guide for ecology and evolution. Trends Ecol. Evol. 24: 127–135 19185386 10.1016/j.tree.2008.10.008

[bib2] Dempster E. R. Lerner I. M., 1950 Heritability of threshold characters. Genetics 35: 212–236 17247344 10.1093/genetics/35.2.212 PMC1209482

[bib3] Eidsvik J. Martino S. Rue H., 2009 Approximate Bayesian inference in spatial generalized linear mixed models. Scand. J. Stat. 36: 1–22

[bib4] Foerster K. Coulson T. Sheldon B. C Pemberton J. M. Clutton-Brock T. H., 2007 Sexually antagonistic genetic variation for fitness in red deer. Nature 447: 1107–1119 17597758 10.1038/nature05912

[bib5] Fong Y. Rue H. Wakefield J., 2010 Bayesian inference for generalized linear mixed models. Biostatistics 11: 397–412 19966070 10.1093/biostatistics/kxp053 PMC2883299

[bib6] Foulley J. L. Gianola D. Im S., 1987 Genetic evaluation of traits distributed as Poisson-binomial with reference to reproductive characters. Theor. Appl. Genet. 73: 870 24241297 10.1007/BF00289392

[bib7] Gelman A., 2006 Prior distributions for variance parameters in hierarchical models. Bayesian Anal. 1: 515–533

[bib8] Gianola D. Fernando R. L., 1986 Bayesian methods in animal breeding theory. J. Anim. Sci. 63: 217–244

[bib9] Goddard M. E. Hayes B. C., 2009 Mapping genes for complex traits in domestic animals and their use in breeding programmes. Nat. Rev. Genet. 10: 381–391 19448663 10.1038/nrg2575

[bib10] Hadfield J. D., 2008 Estimating evolutionary parameters when viability selection is operating. Proc. R. Soc. B. Biol. Sci. 275: 723–734 10.1098/rspb.2007.1013 PMC2596846 18211873

[bib11] Hadfield J. D., 2010 MCMC methods for multi-response generalized linear mixed models: the MCMCglmm R package. J. Stat. Softw. 33: 1–2220808728

[bib12] Hadfield J. D. Wilson A. J. Garant D. Sheldon B. C. Kruuk L. E. B., 2010 The misuse of BLUP in ecology and evolution. Am. Nat. 175: 116–125 19922262 10.1086/648604

[bib13] Hansen T. F. Pélabon C. Houle D., 2011 Heritability is not evolvability. Evol. Biol. 38: 258–277

[bib14] Henderson C., 1950 Estimation of genetic parameters. Ann. Math. Stat. 21: 309–310

[bib15] Jensen H. Steinsland I. Ringsby T. H. Sæther B.-E., 2008 Evolutionary dynamics of a sexual ornament in the house sparrow (*Passer domesticus*): the role of indirect selection within and between sexes. Evolution 62: 1275–1293 18384654 10.1111/j.1558-5646.2008.00395.x

[bib16] Jones J. S., 1987 The heritability of fitness: bad news for ‘good genes’? Trends Ecol. Evol. 2: 35–38 21227813 10.1016/0169-5347(87)90096-6

[bib17] Kruuk L., 2004 Estimating genetic parameters in natural populations using the ’animal model’. Phil. Trans. R. Soc. B 359: 874–890 10.1098/rstb.2003.1437 PMC1693385 15306404

[bib18] Kruuk L. E. B. Slate J. Wilson A. J., 2008 New answers for old questions: The evolutionary quantitative genetics of wild animal populations. Annu. Rev. Ecol. Evol. Syst. 39: 525–548

[bib19] Lande R. Arnold S. J., 1983 The measurement of selection on correlated characters. Evolution 37: 1210–1226 10.1111/j.1558-5646.1983.tb00236.x 28556011

[bib20] Lunn D. Thomas A. Best N. Spiegelhalter D., 2000 WinBUGS—a Bayesian modelling framework: concepts, structure, and extensibility. Stat. Comput. 10: 325–337

[bib21] Lynch M. Walsh B., 1998 Genetics and Analysis of Quantitative Traits. Sinauer Associates, Sunderland, MA.

[bib22] Martino S. Akerkar R. Rue H., 2011 Approximate Bayesian inference for survival models. Scand. J. Stat. 38: 514–528

[bib23] Matos C. Thomas D. L. Gianola D. Perez-Enciso M. Young L. D., 1997 Genetic analysis of discrete reproductive traits in sheep using linear and nonlinear models: II. Goodness of fit and predictive ability. J. Anim. Sci. 75: 88–94 9027552 10.2527/1997.75188x

[bib24] Merilä J. Sheldon B. C., 2000 Lifetime reproductive success and heritability in nature. Am. Nat. 155: 301–310 10718727 10.1086/303330

[bib25] Meyer K., 1991 Estimating variances and covariances for multivariate animal models by restricted maximum likelihood. Genet. Sel. Evol. 23: 67–83

[bib26] O’Hara R. B. Cano J. M. Ovaskainen O. Teplitsky C. Alho J. S., 2008 Bayesian approaches in evolutionary quantitative genetics. J. Evol. Biol. 21: 949–957 18373658 10.1111/j.1420-9101.2008.01529.x

[bib27] Ovaskainen O. Cano J. M. Merilä J., 2008 A Bayesian framework for comparative quantitative genetics. Proc. R. Soc. B Biol. Sci. 275: 669–678 10.1098/rspb.2007.0949 PMC2596838 18211881

[bib28] Postma E., 2006 Implications of the difference between true and predicted breeding values for the study of natural selection and micro-evolution. J. Evol. Biol. 19: 309–320 16599906 10.1111/j.1420-9101.2005.01007.x

[bib29] Pärn H. Jensen H. Ringsby T. H. Sæther B.-E., 2009 Sex-specific fitness correlates of dispersal in a house sparrow metapopulation. J. Anim. Ecol. 78: 1216–1225 19674180 10.1111/j.1365-2656.2009.01597.x

[bib30] Quaas R., 1976 Computing the diagonal elements and inverse of a large numerator relationship matrix. Biometrics 32: 949–953

[bib31] Ringsby T. H. Sæther B.-E. Tufto J. Jensen H. Solberg E., 2002 Asynchronous spatiotemporal demography of a house sparrow metapopulation in a correlated environment. Ecology 83: 561–569

[bib32] Roos M. Held L., 2011 Sensitivity analysis in bayesian generalized linear mixed models for binary data. Bayesian Anal. 6: 259–278

[bib33] Rue H. Held L., 2005 Gaussian Markov Random Fields: Theory and Applications. Chapman & Hall, London

[bib34] Rue H. Martino S., 2007 Approximate Bayesian inference for hierarchical Gaussian Markov random field models. J. Statist. Plann. Inference 137: 3177–3192

[bib35] Rue H. Martino S. Chopin N., 2009 Approximate Bayesian inference for latent Gaussian models using integrated nested Laplace approximations. J. R. Stat. Soc., B 71: 319–392

[bib36] Schrödle B. Held L. Riebler A. Danuser J., 2011 Using integrated nested Laplace approximations for the evaluation of veterinary surveillance data from Switzerland: a case-study. J. Roy. Stat. Soc. C-App. 60: 261–279

[bib37] Simm G., 1998 Genetic Improvement of Cattle and Sheep. Farming Press, Ipswich, U.K.

[bib38] Skrondal A. Rabe-Hesketh S., 2004 *Generalized Latent Variable Modeling: Multilevel*, *Longitudinal*, *and Structural Equation Models*. Chapman & Hall/CRC, Boca Raton, FL

[bib39] Sorensen D. Gianola D., 2002 *Likelihood*, *Bayesian and MCMC Methods in Quantitative Genetics*. Springer-Verlag, New York

[bib40] Sorensen D. Wang C. S. Jensen J. Gianola D., 1994 Bayesian analysis of genetic change due to selection using Gibbs sampling. Genet. Sel. Evol. 26: 333–360

[bib41] Spiegelhalter D. J. Best N. G. Carlin B. P. van der Linde A., 2002 Bayesian measures of model complexity and fit (with discussion). J. R. Stat. Soc. B. 64: 583–639

[bib42] Steinsland I. Jensen H., 2010 Utilizing Gaussian Markov random field properties of Bayesian animal models. Biometrics 66: 763–771 19817739 10.1111/j.1541-0420.2009.01336.x

[bib43] Tempelman R. J. Gianola D., 1994 Assessment of a Poisson animal model for embryo yield in a simulated multiple ovulation-embryo transfer scheme. Genet. Sel. Evol. 26: 263–290

[bib44] Van De Wiel M. A. Leday G. G., 2013 Bayesian analysis of RNA sequencing data by estimating multiple shrinkage priors. Biostatistics 14: 113–128 22988280 10.1093/biostatistics/kxs031

[bib45] Vazquez A. I. Gianola D. Bates D. Weigel K. A. Heringstad B., 2009 Assessment of Poisson, logit, and linear models for genetic analysis of clinical mastitis in Norwegian Red cows. J. Dairy Sci. 92: 739–748 19164686 10.3168/jds.2008-1325

[bib46] Visscher P. M. Hill W. G. Wray N. R., 2008 Heritability in the genomics era— concepts and misconception. Nat. Rev. Genet. 9: 255–266 18319743 10.1038/nrg2322

[bib47] Wilson A. J. Réale D. Clements M. N. Morrissey M. M. Postma E., 2009 An ecologist's guide to the animal model. J. Anim. Ecol. 79: 13–26 20409158 10.1111/j.1365-2656.2009.01639.x

[bib48] Yang W. Tempelman R. J., 2012 A Bayesian antedependence model for whole genome prediction. Genetics 190: 1491–1501 22135352 10.1534/genetics.111.131540 PMC3316658

