# Supplementary material for: Animal Models and Integrated Nested Laplace Approximations
Source: G3 (Bethesda). 2013 Aug 1;3(8):1241–51. doi: 10.1534/g3.113.006700 (PMC3737164; doi:10.1534/g3.113.006700)
Supplement: Supporting Information [file supp_3_8_1241__index.html]

Animal Models and Integrated Nested Laplace Approximations — Supporting Information 

# Animal Models and Integrated Nested Laplace Approximations

## Supporting Information for Holand *et al.*, 2013

**Files in this Data Supplement:**

- Supporting Information - Figures S1-S6, Files S1-S5, and Table S1 (PDF, 901 KB)
- Figure S1 - Histogram showing phenotypic bill depth observations for house sparrows in northern Norway, indicating a Gaussian distribution. (PDF, 50 KB)
- Figure S2 - Comparison of INLA and MCMC (PDF, 383 KB)
- Figure S3 - Prior sensitivity analyses for synthetic Gaussian, binary, Binomial, and Poisson case studies (PDF, 99 KB)
- Figure S4 - Comparison of INLA and MCMC (PDF, 401 KB)
- Figure S5 - Posterior of difference in mean breeding values for bill depth between cohorts 1993 and 2002 in house sparrows in northern Norway (PDF, 52 KB)
- Figure S6 - Histgram showing observed lifetime reproductive sucess (LRS) relative to the lifespan (LRS/lifespan) in house sparrows in northern Norway, indicating a zero-inflated Poisson distribution (PDF, 47 KB)
- File S1 - Model formulations for Gaussian animal model (PDF, 124 KB)
- File S2 - Prior sensitivity analysis for synthetic datasets (PDF, 102 KB)
- File S3 - R code for synthetic data using the R package AnimalINLA (PDF, 140 KB)
- File S4 - R code for random effects in INLA (PDF, 95 KB)
- Table S1 - Inference from INLA for synthetic Poisson data (PDF, 132 KB)
- File S5 - Datasets (.zip, 26 KB)
