# Supplementary material for: Animal Models and Integrated Nested Laplace Approximations
Source: G3 (Bethesda). 2013 Aug 1;3(8):1241–51. doi: 10.1534/g3.113.006700 (PMC3737164; doi:10.1534/g3.113.006700)
Supplement: Supporting Information [file supp_g3.113.006700_006700SI.pdf]

Animal models and Integrated Nested Laplace Approximations

Anna Marie Holand[*,1], Ingelin Steinsland[**], Sara Martino[**,§], Henrik Jensen[*]

[*]Centre for Biodiversity Dynamics, Department of Biology, NTNU, NO-7491 Trondheim, Norway, [**]Centre for Biodiversity Dynamics, Department of Mathematical Sciences, NTNU, NO-7491 Trondheim, Norway, [§]Current address: SINTEF Energi AS, 7465 Trondheim, Norway

[1]Corresponding author: Anna Marie Holand, Centre for Biodiversity Dynamics, Department of Biology, Norwegian University of Science and Technology, NO-7491 Trondheim, Norway, Email: Anna.Marie.Holand@nt.ntnu.no

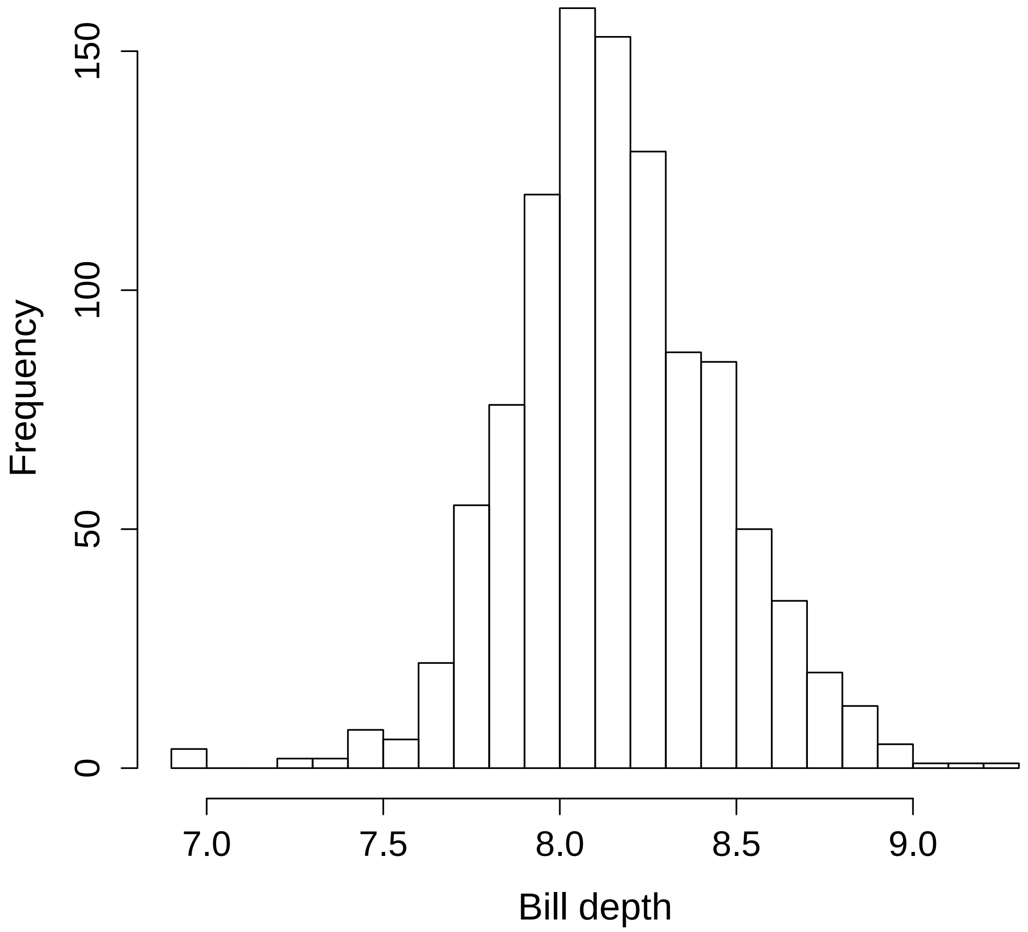

Figure S1 Histogram showing phenotypic bill depth observations for house sparrows in northern Norway, indicating a Gaussian distribution.

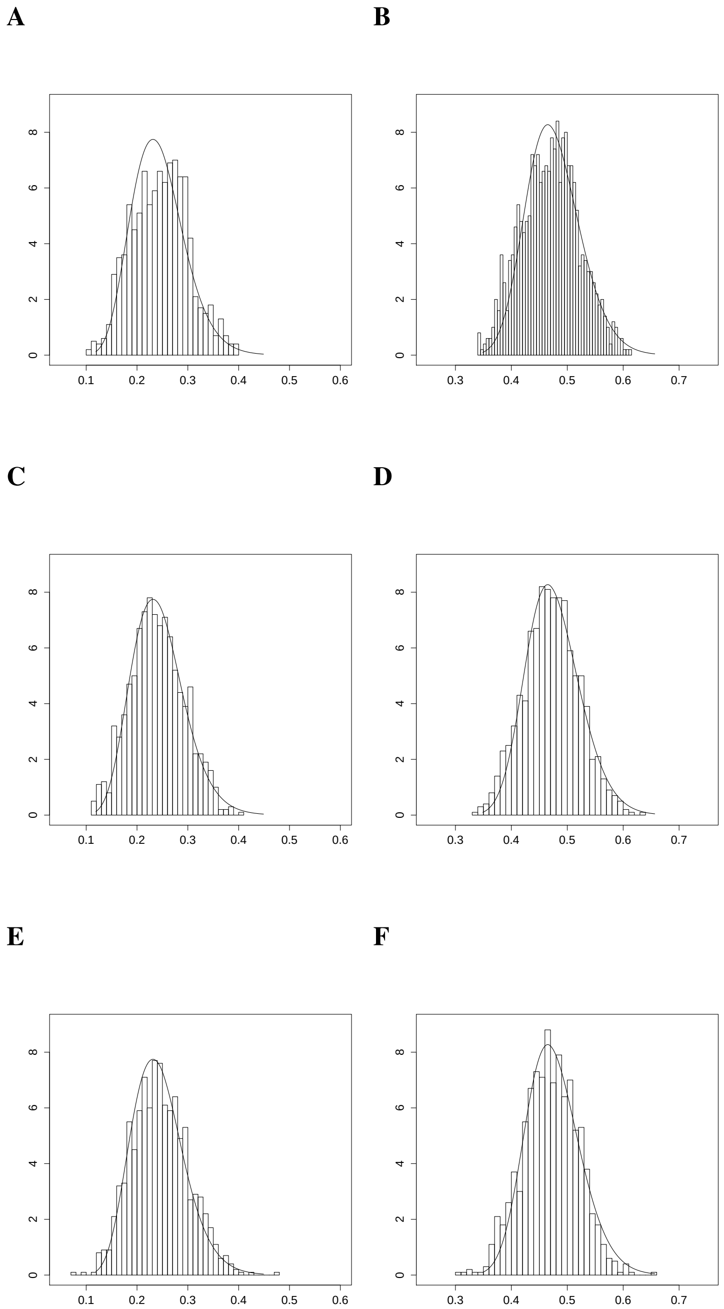

Figure S2 Comparison of INLA and MCMC. INLA (solid line) and MCMC estimates (histogram) for different number of iterations for MCMC for the posterior marginal of σ_u^2 and σ_e^2 for the bill depth of house sparrows in northern Norway: 10000 iterations **(A)** σ_u^2 and **(B)** σ_e^2, 100000 iterations **(C)** σ_u^2 and **(D)** σ_e^2, 200000 iterations **(E)** σ_u^2 and **(F)** σ_e^2. INLA used 7 seconds and MCMC used 51 seconds, 8.4 minutes and 17 minutes, respectively.

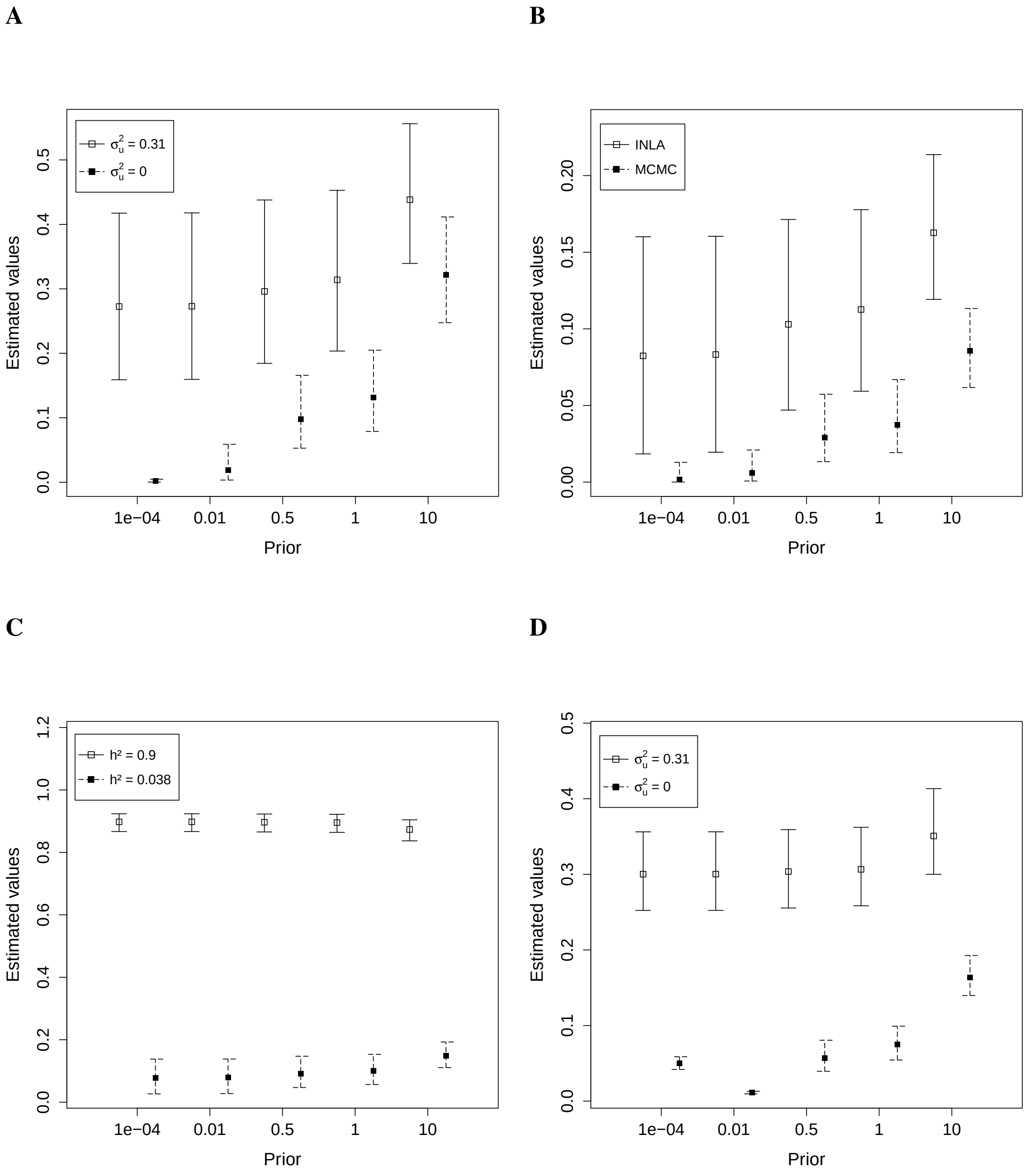

Figure S3 Prior sensitivity analyses for synthetic Gaussian, binary, Binomial, and Poisson case studies. Posterior mean with 95% credible interval for prior InvGamma(a, b), where $a = b$ is equal to 0.0001, 0.01, 0.5, 1 and 10, respectively (note that estimates are shifted relative to the x-axis for clarity). **(A)** Gaussian data for $\sigma_u^2 = 0$ (filled squares, dashed line) and $\sigma_u^2 = 0.31$ (open squares, solid line) for INLA, **(B)** binary data for $h^2 = 0$, INLA (open squares, solid line) and MCMC (filled squares, dashed line), **(C)** Binomial data for $h^2 = 0.9$ (open squares, solid line) and $h^2 = 0.038$ (filled squares, dashed line) for INLA, **(D)** zero-inflated Poisson data for $\sigma_u^2 = 0.31$ (open squares, solid line) and $\sigma_u^2 = 0$ (filled squares, dashed line) for INLA.

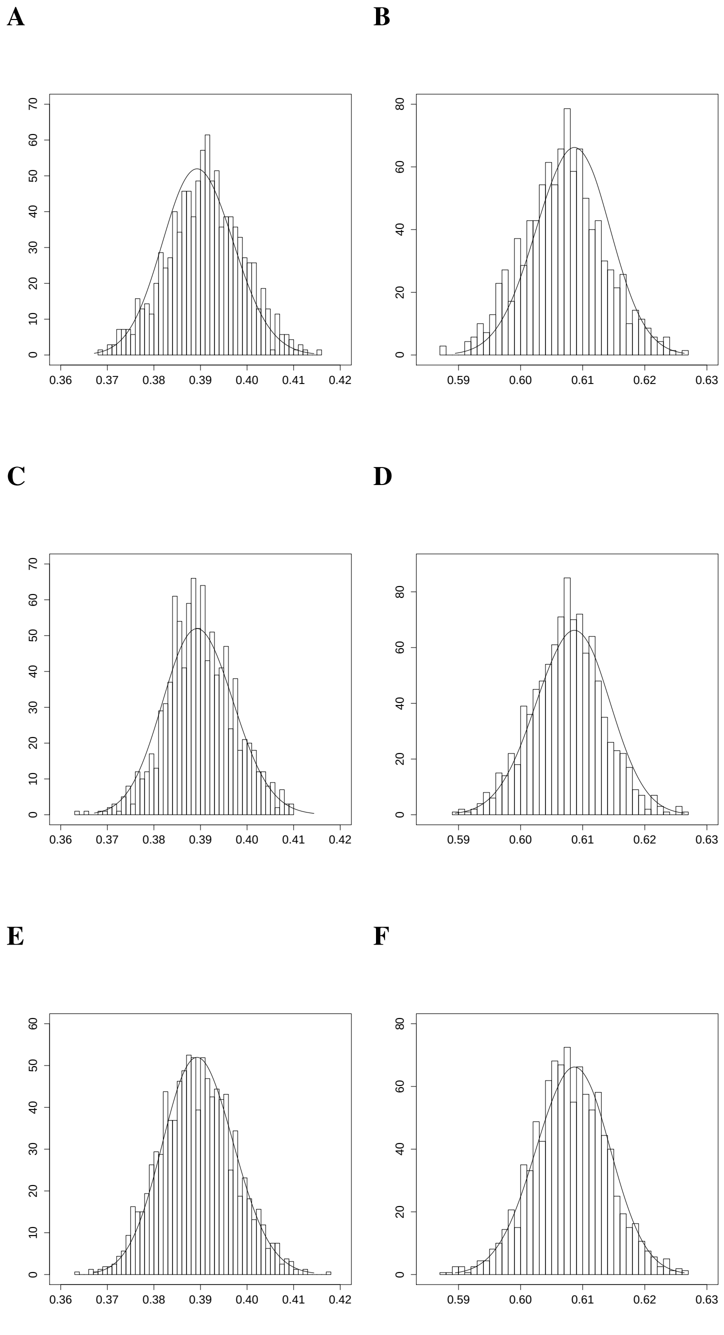

Figure S4 Comparison of INLA and MCMC. INLA (solid line) and MCMC estimates (histogram) for different number of iterations for MCMC for the posterior marginal of σ_u^2 and σ_e^2 for a large synthetic pedigree and simulated dataset of $n_p = 100072$ individuals: 10000 iterations **(A)** σ_u^2 and **(B)** σ_e^2, 100000 iterations **(C)** σ_u^2 and **(D)** σ_e^2, 500000 iterations **(E)** σ_u^2 and **(F)** σ_e^2. INLA used 7.4 minutes and MCMC used 29 minutes, 3.6 hours and 17.9 hours, respectively.

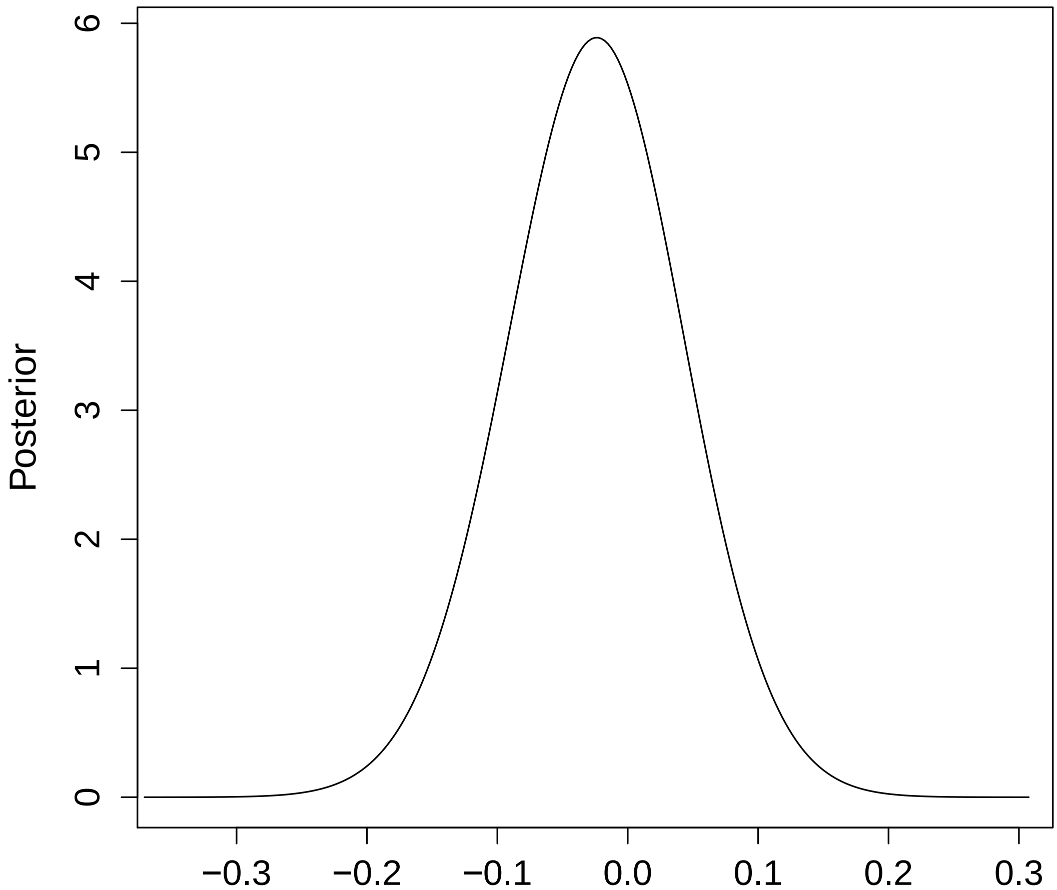

Figure S5 Posterior of difference in mean breeding values for bill depth between cohorts 1993 and 2002 in house sparrows in northern Norway.

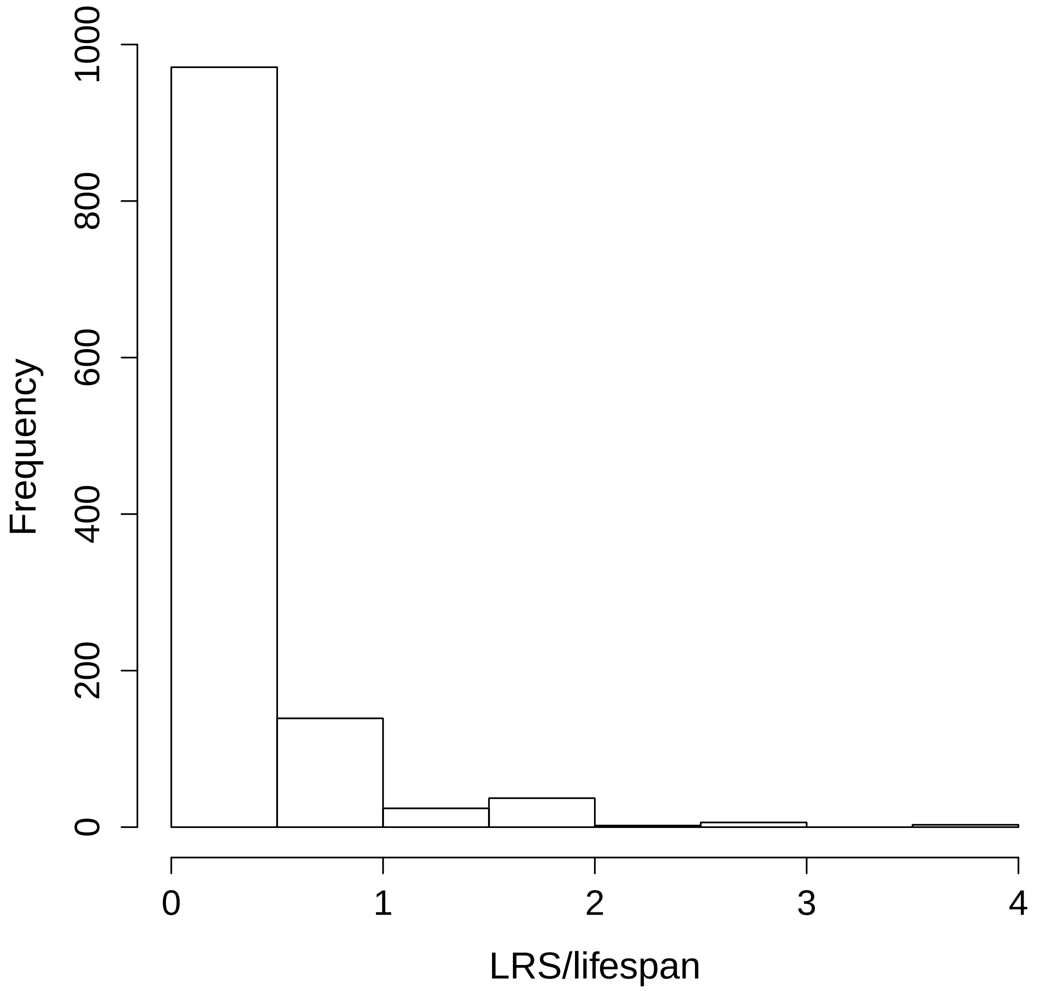

Figure S6 Histgram showing observed lifetime reproductive sucess (LRS) relative to the lifespan (LRS/lifespan) in house sparrows in northern Norway, indicating a zero-inflated Poisson distribution.

Table S1 Inference from INLA for synthetic Poisson data. Simulated under model $\mathbf{y_i} \mid \lambda_\mathbf{i} \sim \mathbf{Pois}(\mathbf{n_i}, \lambda_\mathbf{i})$, $\eta_\mathbf{i} = \mathbf{log}(\lambda_\mathbf{i}) = \beta_\mathbf{0} + \mathbf{u_i}$, with $\beta_\mathbf{0} = \mathbf{0}$, levels of σ_u^2 ranging from 0 to 1, and missing pattern similar to the house sparrow Poisson case study. $\hat{\sigma}_\mathbf{u}^\mathbf{2}$ is the posterior mean additive genetic variance with standard deviations (sd), and 95% credible interval (CI).

$\boldsymbol{\sigma_u^2}$	$\hat{\sigma}_u^2$ (sd)	95% CI
**0**	0.08 (0.02)	(0.05,0.13)
**0.05**	0.09 (0.02)	(0.05,0.13)
**0.1**	0.12 (0.03)	(0.07,0.18)
**0.15**	0.14 (0.03)	(0.09,0.21)
**0.2**	0.20 (0.04)	(0.13,0.27)
**0.3**	0.33 (0.05)	(0.25,0.43)
**0.4**	0.43 (0.05)	(0.33,0.54)
**0.5**	0.53 (0.06)	(0.43,0.65)
**0.6**	0.60 (0.06)	(0.49,0.73)
**0.7**	0.68 (0.06)	(0.56,0.81)
**0.8**	0.84 (0.08)	(0.69,1.00)
**0.9**	0.91 (0.08)	(0.77, 1.08)
**1**	0.99 (0.09)	(0.83,1.17)

File S1

Model formulations for Gaussian animal model

A Gaussian animal model can be formulated in two alternative ways, both fitting the INLA framework.

Model formulation 1 (MF1): Likelihood $y_i|\eta_i \sim \mathcal{N}(\eta_i, \sigma_e^2)$ and latent field $\eta_i = \beta_0 + z_i^T\beta + u_i + \epsilon_i$, where the variance of ϵ is fixed to a small value.

Model formulation 2 (MF2): Likelihood $y_i|\eta_i \sim \mathcal{N}(\eta_i, \sigma_{small}^2)$, i.e. the variance of the likelihood is fixed to a small value, and latent field $\eta_i = \beta_0 + z_i^T\beta + u_i + \epsilon_i$, where the variance of ϵ is σ_e^2. The σ_{small}^2 can be interpreted as measurement uncertainty.

When estimating the narrow sense heritability, h^2, in the Gaussian case, we use model formulation MF2, which out of convenience is parametrized with (σ_u^2, h^2) instead of (σ_u^2, σ_e^2). Further, (σ_u^2, h^2) is given a prior such that it corresponds to the prior of (σ_u^2, σ_e^2), hence, the same prior under two different parametrizations.

The DIC is based on evaluating the likelihood, and is not invariant with respect to parametrization (Spiegelhalter *et al.* 2002). Using model formulation MF2, i.e. a fixed small variance for the likelihood does not work numerically; almost all models get the same DIC to the precision given by INLA. So if DIC needs to be calculated the animal model has to be formulated in an alternative way (in the INLA framework), where the variance of ϵ is fixed to a small value in the latent field, i.e. using MF1. Both model formulations coincide if the same priors are used for the hyper-parameters $(\beta, \sigma_e^2, \sigma_u^2)$, and are latent Gaussian fields with only two non-Gaussian parameters, namely $\boldsymbol{\theta} = (\sigma_u^2, \sigma_e^2)$. For MF1 $\boldsymbol{\epsilon}$ can be omitted from the model. It is included here to be consistent with MF2. Both model formulations have their numerical advantages depending on the aim of the analysis. However, we have to be cautious which model formulation we use depending on the purpose of the analysis.

To summarize, when u_i, $\sum_{i \in C} w_i u_i$, β or σ_u^2 is of interest both MF1 and MF2 might be used. If σ_e^2 or DIC is the aim of the analysis MF1 has to be used, while MF2 with parametrization (σ_e^2, h^2) has to be used if h^2 is of interest. Hence we might have to fit two (INLA) models to get all estimates of interest.

Literature Cited

Spiegelhalter, D. J., N. G. Best, B. P. Carlin, and A. van der Linde, 2002 Bayesian measures of model complexity and fit (with discussion). J. Roy. Stat. Soc. B 64: 583–639.

File S2

Prior sensitivity analysis for synthetic datasets

To test for prior sensitivity we do a sensitivity analysis for several synthetic datasets similar to those in **Synthetic case studies section**. The house sparrow pedigree with Gaussian, binary, binomial and Poisson likelihoods are used. Each dataset is analyzed with five different priors for σ_u^2 and, when relevant, σ_ϵ^2; $InvGamma(a, b)$ with $a = b = \{0.0001, 0.01, 0.5, 1, 10\}$. These priors range from uninformative priors to very informative; $InvGamma(10, 10)$ has expected value 1.1 and a standard deviation of 0.37. The results from the sensitivity analyses are visualized in Figure S3.

(A) shows results for two synthetic Gaussian datasets, simulated under model $y_i|\mu_i, \sigma_e^2 \sim \mathcal{N}(\mu_i, \sigma_e^2), \eta_i = \mu_i = \beta_0 + u_i$, with $\beta_0 = 0$ and $\sigma_u^2 + \sigma_\epsilon^2 = 1$ for $i)$ $\sigma_u^2 = 0$ and $ii)$ $\sigma_u^2 = 0.31$. The same missing data structure as in the house sparrow Gaussian case study is imposed giving 1025 individuals in the dataset. Inference is done with INLA. We find that with no heritability ($\sigma_u^2 = 0$) the results are very prior sensitive, while with a heritability of $h^2 = \sigma_u^2 = 0.31$ only the most informative prior changes the inference considerably.

(B) shows results for synthetic binary dataset with observations for all the individuals in the pedigree. The data are simulated from a model with logit link, $\eta_i = \beta_0 + u_i$ with $\beta_0 = 0$ and no genetic component ($\sigma_u^2 = 0$). As we in **Synthetic Binomial case study section** experienced problems using INLA in the binary case, the inference is done with both INLA and MCMC. From the MCMC results we find that the inference is prior sensitive, and also that the systematic errors for INLA are prior sensitive.

(C) shows results for two synthetic binomial datasets. In both datasets the number of trials n_i is as in the house sparrow breeding season success dataset, and also the missing patterns coincide with this. A logit link is used and $\eta_i = \beta_0 + u_i$ with $\beta_0 = 0$. We have a case with high heritability; $i)$ $h^2 = 0.9$ and one with low $ii)$ $h^2 = 0.038$ (or $\sigma_u^2 = 0.13$ as estimated from the breeding season success dataset). Analyses are done using INLA. We find that neither case is very prior sensitive.

(D) shows results for two synthetic zero-inflated Poisson datasets. They are simulated under model $y_i \mid \lambda_i \sim Pois(n_i, \lambda_i), \eta_i = log(\lambda_i) = \beta_0 + u_i$ with $\beta_0 = 0$, with missing pattern as in the house sparrow Poisson case study, and with no heritability ($h^2 = \sigma_u^2 = 0$) and moderate heritability ($\sigma_u^2 = 0.31$). Inference is done with INLA. The results are very prior sensitive for the dataset without heritability, while only the most informative prior gives any considerable difference for the dataset simulated with

$\sigma_u^2 = 0.31$.

File S3

R code for synthetic data using the R package `AnimalINLA`

R code for simulating data with same dependency as the real pedigree, where the sparse structure matrix `Cmatrix` is obtained from $\boldsymbol{A}^{-1}$ calculated in the R package `AnimalINLA` (`www.r-inla.org/related-projects/animalinla`).

We simulated data with different values of σ_u^2 =`var.u` and σ_e^2 =`var.e` with the function simulate.breeding.values:

```
Simulation code for breeding value:
##need the package "spam"
install.packages("spam")

inla.complete.Cmatrix <- function(C)
{
    idx = (C$i != C$j)
    return (list(i=c(C$i, C$j[idx]), j=c(C$j, C$i[idx]),
                 values=c(C$values, C$values[idx])))
}
simulate.breeding.values <- function(Cmatrix, varu, nsamples = 1)
{
    library(spam)
    prec = 1/varu
    Comp = inla.complete.Cmatrix(Cmatrix)
    S = spam(x = list(i = Comp$i, j = Comp$j, values =
                                   Comp$values))
    Q = prec * S
    breeding = rmvnorm.prec(nsamples,mu=rep(0, nrow(Q)), Q)
        breeding = as.vector(breeding)
```

```
}

##define the sparse-matrix from the relationship matrix
##computed in compute.Ainverse(), used in simulate.breeding.values()
Cmatrix = list(i= xx$Ainverse[,1],j = xx$Ainverse[,2], values =xx$Ainverse[,3])
```

Synthetic Gaussian case study

```
library(AnimalINLA)
data(sparrowpedigree)

##Run AnimalINLA
xx=compute.Ainverse(sparrowpedigree)

##number of individuals in the pedigree
Nbird = dim(sparrowpedigree)[1]
## choose the values of the hyperparameters
var.u = 0.6
var.e = 0.4

## simulate the breeding values and the environemental effect
breeding = simulate.breeding.values(Cmatrix, var.u)
env = rnorm(Nbird, mean = 0, sd = sqrt(var.e))

## compute the trait
trait =  breeding + env

## make the data frame
data = data.frame(y=trait,u=1:Nbird)
```

```
##Run AnimalINLA
gauss=animal.inla(response=y, genetic=c("u"),
                  Ainverse =sparseMatrix(i=xx$Ainverse[,1],
                  j=xx$Ainverse[,2],x=xx$Ainverse[,3]),
                  data=data, type.data="gaussian",
                  dic=TRUE,sigma.e=TRUE)

##hyperparameteres
gauss$summary.hyperparam
```

Synthetic Binomial case study

```
library(AnimalINLA)
data(sparrowpedigree)

##need the package "boot"
install.packages("boot")
library(boot)

## numbers of individuals in the pedigree
Nbird = dim(pedigree)[1]

## set the value for the hyperparameter, where beta0 is the intercept
var.u = 0.3
beta0 = 1

## set the number of trials
Ntrials = sample(1:9, 3574 , replace=T)

## simulate breeding values
```

```
breeding = simulate.breeding.values(Cmatrix, var.u)
eta =  beta0 + breeding
p = inv.logit(eta)

## simulate the trait
trait = rbinom(Nbird, Ntrials, p)

data = data.frame(y = trait,u = 1:Nbird,
                  Ntrial = Ntrials)

##Run AnimalINLA
xx=compute.Ainverse(sparrowpedigree)

bin=animal.inla(response=y, genetic=c("u"),
                Ntrials = Ntrial,
                Ainverse =sparseMatrix(i=xx$Ainverse[,1],
                j=xx$Ainverse[,2],x=xx$Ainverse[,3]),
                data=data,type.data="binomial",
                dic=TRUE)

##hyperparameteres
bin$summary.hyperparam
```

Synthetic Poisson case study

```
library(AnimalINLA)
data(sparrowpedigree)

##number of individuals in the pedigree
Nbird = dim(sparrowpedigree)[1]
```

```
## choose the values of the hyperparameters
var.u =  0.7
beta0 = 1

##Run AnimalINLA
breeding = simulate.breeding.values(Cmatrix, var.u)

## compute the trait
eta = beta0 + breeding
lambda=exp(eta)
trait=rpois(Nbird,lambda)

## make the data frame
data = data.frame(y=trait,u=1:Nbird,n=rep(1,Nbird))

##Run AnimalINLA
xx=compute.Ainverse(sparrowpedigree)

pois=animal.inla(response="y", genetic=c("u"),
                 Ainverse =sparseMatrix(i=xx$Ainverse[,1],
                 j=xx$Ainverse[,2],x=xx$Ainverse[,3]),
                 E=n,data=data,type.data="poisson",dic=TRUE)

##hyperparameters
pois$summary.hyperparam
```

File S4

R code for random effects in INLA

Including individual as a independent random effect in the latent field is implemented the same way in INLA for all case studies in house sparrow population (Gaussian, binomial and Poisson). Note that in the Gaussian case study we have repeated measurements, i.e. possible several observation for each individual random effect, while in the binomial and Poisson cases there are only one observation for each individual. For the simulated datasets in `AnimalINLA` (only one measurement for each individual);

```
library(AnimalINLA)
library(INLA)
library(Matrix)

data(sparrowpedigree)
xx = compute.Ainverse(sparrowpedigree)
Ainv = xx$Ainverse
map  = xx$map
Cmatrix = sparseMatrix(i=Ainv[,1],j=Ainv[,2],x=Ainv[,3])
```

Gaussian case study:

```
data(sparrowGaussian)
Ndata = dim(sparrowGaussian)[1]

## Mapping the same index number for "Individual" as in Ainv
## The IndexA column is the index in the A inverse matrix
sparrowGaussian$IndexA = rep(0,Ndata)
for(i in 1:Ndata)
  sparrowGaussian$IndexA[i] = which(map[,1]==sparrowGaussian$Individual[i])

#Including an extra column for individual effect
sparrowGaussian$IndexA.2=sparrowGaussian$IndexA
```

```
formula = y ~ f(IndexA,model="generic0", Cmatrix=Cmatrix,
    constr=TRUE,param = c(0.5, 0.5)) +
                      f(IndexA.2,model="iid",param = c(1,0.001),
                      constr=TRUE)
```

y in $formula$ is the trait, i.e bill depth in the case study, *IndexA* and *IndexA.2* is the individuals in the data (these have to be given different names) where *IndexA* is the additive genetic effect and *IndexA.2* is the individual random effect.

the likelihood is implemented in the inla call;

```
model = inla(formula=formula, family="gaussian",
         data=sparrowGaussian,
         control.family=list(hyper = list(theta =
         list(param = c(0.5, 0.5), fixed = FALSE))),
 only.hyperparam =FALSE,control.compute=list(dic=T))

summary(model)
#Example finding the posterior marginal distribution and mean (95% CI)
#for additive genetic variance and individual random variance
sigma.IndexA = inla.marginal.transform(function(x) 1/x,
             model$marginals.hyperpar$"Precision for IndexA")
e.IndexA=inla.expectation(function(x) x, sigma.IndexA)
ci.IndexA=inla.qmarginal(c(0.025, 0.975), sigma.IndexA)

#and posterior marginal distribution and mean (95% CI)
#for individual random variance
sigma.IndexA.2 = inla.marginal.transform(function(x) 1/x,
                model$marginals.hyperpar$"Precision for IndexA.2")
e.IndexA.2=inla.expectation(function(x) x, sigma.IndexA.2)
ci.IndexA.2=inla.qmarginal(c(0.025, 0.975), sigma.IndexA.2)
```

Binomial case study:

```
data(sparrowBinomial)

Ndata = dim(sparrowBinomial)[1]

## Mapping the same index number for "Individual" as in Ainv
## The IndexA column is the index in the A inverse matrix
sparrowBinomial$IndexA = rep(0,Ndata)
for(i in 1:Ndata)
  sparrowBinomial$IndexA[i] = which(map[,1]==sparrowBinomial$Individual[i])

#Including an extra column for individual effect
sparrowBinomial$IndexA.2=sparrowBinomial$IndexA

formula = y ~ f(IndexA,model="generic0", Cmatrix=Cmatrix,
    constr=TRUE,param = c(0.5, 0.5)) +
                      f(IndexA.2,model="iid",param = c(1,0.001),
                      constr=TRUE)
```

y in $formula$ is the trait, i.e number of years individuals produced at least one recruit in the case study, *IndexA* and *IndexA.2* is the individuals in the data (these have to be given different names) where *IndexA* is the additive genetic effect and *IndexA.2* is the individual random effect.

The likelihood is implemented in the inla call;

```
model = inla(formula=formula , family="binomial", data=sparrowBinomial,
 Ntrials=Ntrial,
         only.hyperparam = FALSE,control.compute=list(dic=T))
```

Ntrial is the number of trials, i.e the number of breeding seasons individuals were alive during the study period.

```
summary(model)
#Example finding the posterior marginal distribution and mean (95% CI) for
#additive genetic variance and individual random variance
sigma.IndexA = inla.marginal.transform(function(x) 1/x,
               model$marginals.hyperpar$"Precision for IndexA")
e.IndexA=inla.expectation(function(x) x, sigma.IndexA)
ci.IndexA=inla.qmarginal(c(0.025, 0.975), sigma.IndexA)

#and posterior marginal distribution and mean (95% CI)
#for individual random variance
sigma.IndexA.2 = inla.marginal.transform(function(x) 1/x,
                model$marginals.hyperpar$"Precision for IndexA.2")
e.IndexA.2=inla.expectation(function(x) x, sigma.IndexA.2)
ci.IndexA.2=inla.qmarginal(c(0.025, 0.975), sigma.IndexA.2)
```

Poisson case study:

```
data(sparrowPoisson)

Ndata = dim(sparrowPoisson)[1]

## Mapping the same index number for "Individual" as in Ainv
## The IndexA column is the index in the A inverse matrix
sparrowPoisson$IndexA = rep(0,Ndata)
for(i in 1:Ndata)
sparrowPoisson$IndexA[i] = which(map[,1]==sparrowPoisson$Individual[i])

#Including an extra column for individual effect
```

```
sparrowPoisson$IndexA.2=sparrowPoisson$IndexA

formula = y ~ f(IndexA,model="generic0", Cmatrix=Cmatrix,
    constr=TRUE,param = c(0.5, 0.5)) +
                      f(IndexA.2,model="iid",param = c(1,0.001),
                      constr=TRUE)
```

y in $formula$ is the trait, i.e total number of recruits individuals produced in the study period in the case study, *IndexA* and *IndexA.2* is the individuals in the data (these have to be given different names) where *IndexA* is the additive genetic effect and *IndexA.2* is the individual random effect.

The likelihood is implemented in the inla call;

```
model = inla(formula=formula,
              family="zeroinflatedpoisson1",
              #family="poisson" ,
              data=sparrowPoisson,
              E=n,
              only.hyperparam = FALSE,
              control.compute=list(dic=TRUE))
```

E is the exposure, i.e. the number of breeding seasons individuals were alive during the study period in the case study.

```
summary(model)
#Example finding the posterior marginal distribution and mean (95% CI)
#for additive genetic variance and individual random variance
sigma.IndexA = inla.marginal.transform(function(x) 1/x,
                model$marginals.hyperpar$"Precision for IndexA")
e.IndexA=inla.expectation(function(x) x, sigma.IndexA)
ci.IndexA=inla.qmarginal(c(0.025, 0.975), sigma.IndexA)

#and posterior marginal distribution and mean (95% CI)
```

```
#for individual random variance
sigma.IndexA.2 = inla.marginal.transform(function(x) 1/x,
               model$marginals.hyperpar$"Precision for IndexA.2")
e.IndexA.2=inla.expectation(function(x) x, sigma.IndexA.2)
ci.IndexA.2=inla.qmarginal(c(0.025, 0.975), sigma.IndexA.2)
```
