# Supplementary material for: Animal Models and Integrated Nested Laplace Approximations
Source: G3 (Bethesda). 2013 Aug 1;3(8):1241–51. doi: 10.1534/g3.113.006700 (PMC3737164; doi:10.1534/g3.113.006700)
Supplement: Supporting Information [file supp_g3.113.006700_FigureS1.pdf]

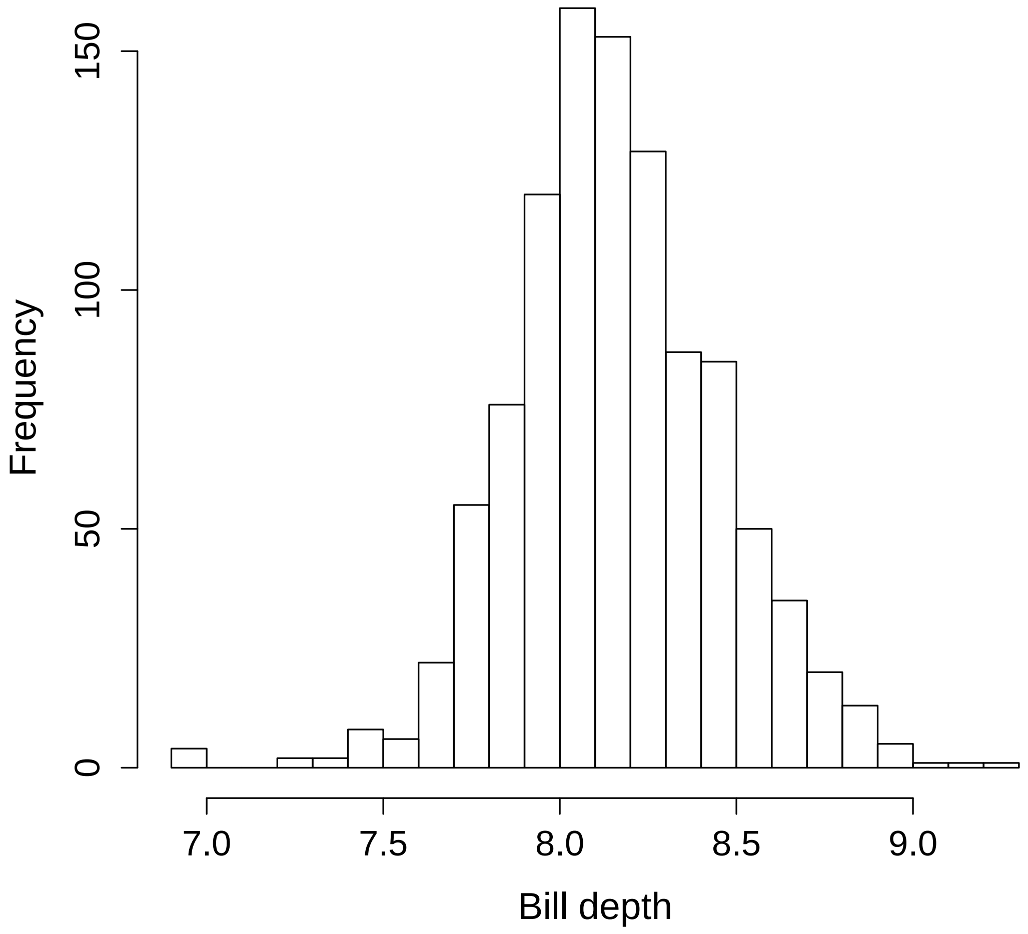

Figure S1 Histogram showing phenotypic bill depth observations for house sparrows in northern Norway, indicating a Gaussian distribution.
