# Supplementary material for: Animal Models and Integrated Nested Laplace Approximations
Source: G3 (Bethesda). 2013 Aug 1;3(8):1241–51. doi: 10.1534/g3.113.006700 (PMC3737164; doi:10.1534/g3.113.006700)
Supplement: Supporting Information [file supp_g3.113.006700_FigureS2.pdf]

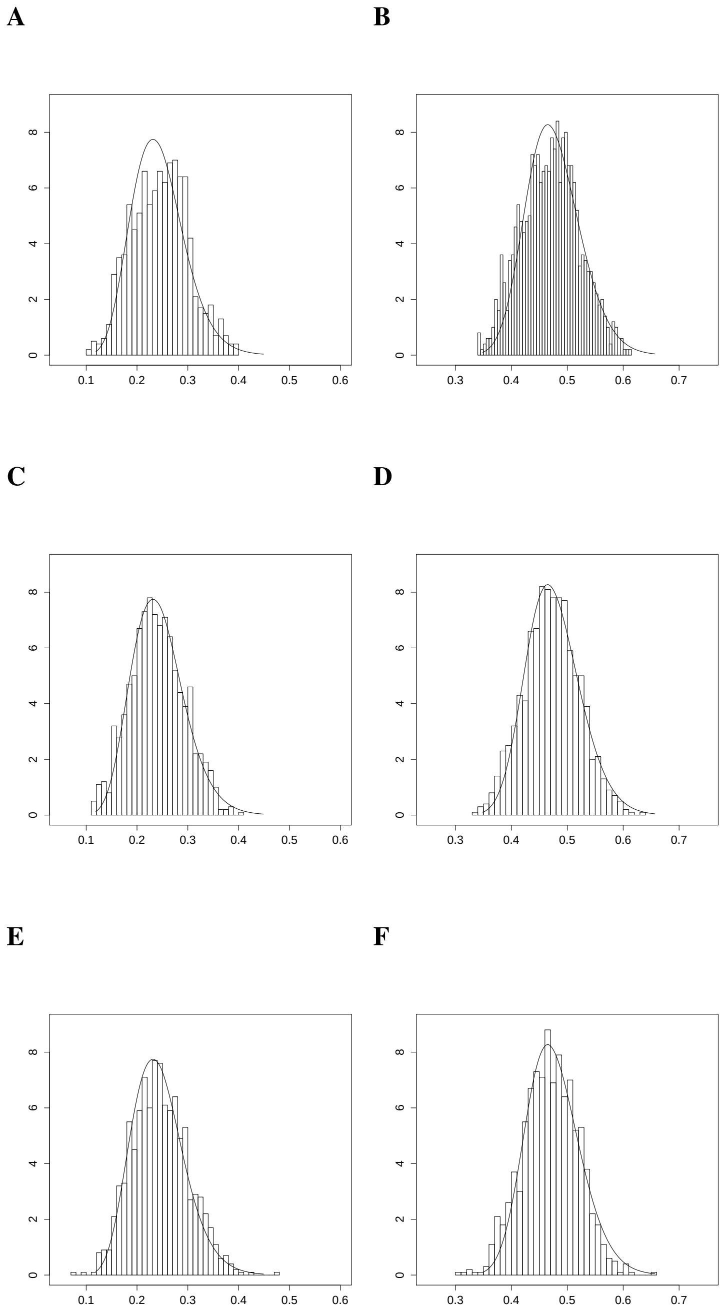

Figure S2 Comparison of INLA and MCMC. INLA (solid line) and MCMC estimates (histogram) for different number of iterations for MCMC for the posterior marginal of σ_u^2 and σ_e^2 for the bill depth of house sparrows in northern Norway: 10000 iterations **(A)** σ_u^2 and **(B)** σ_e^2, 100000 iterations **(C)** σ_u^2 and **(D)** σ_e^2, 200000 iterations **(E)** σ_u^2 and **(F)** σ_e^2. INLA used 7 seconds and MCMC used 51 seconds, 8.4 minutes and 17 minutes, respectively.
