# Supplementary material for: Animal Models and Integrated Nested Laplace Approximations
Source: G3 (Bethesda). 2013 Aug 1;3(8):1241–51. doi: 10.1534/g3.113.006700 (PMC3737164; doi:10.1534/g3.113.006700)
Supplement: Supporting Information [file supp_g3.113.006700_FigureS3.pdf]

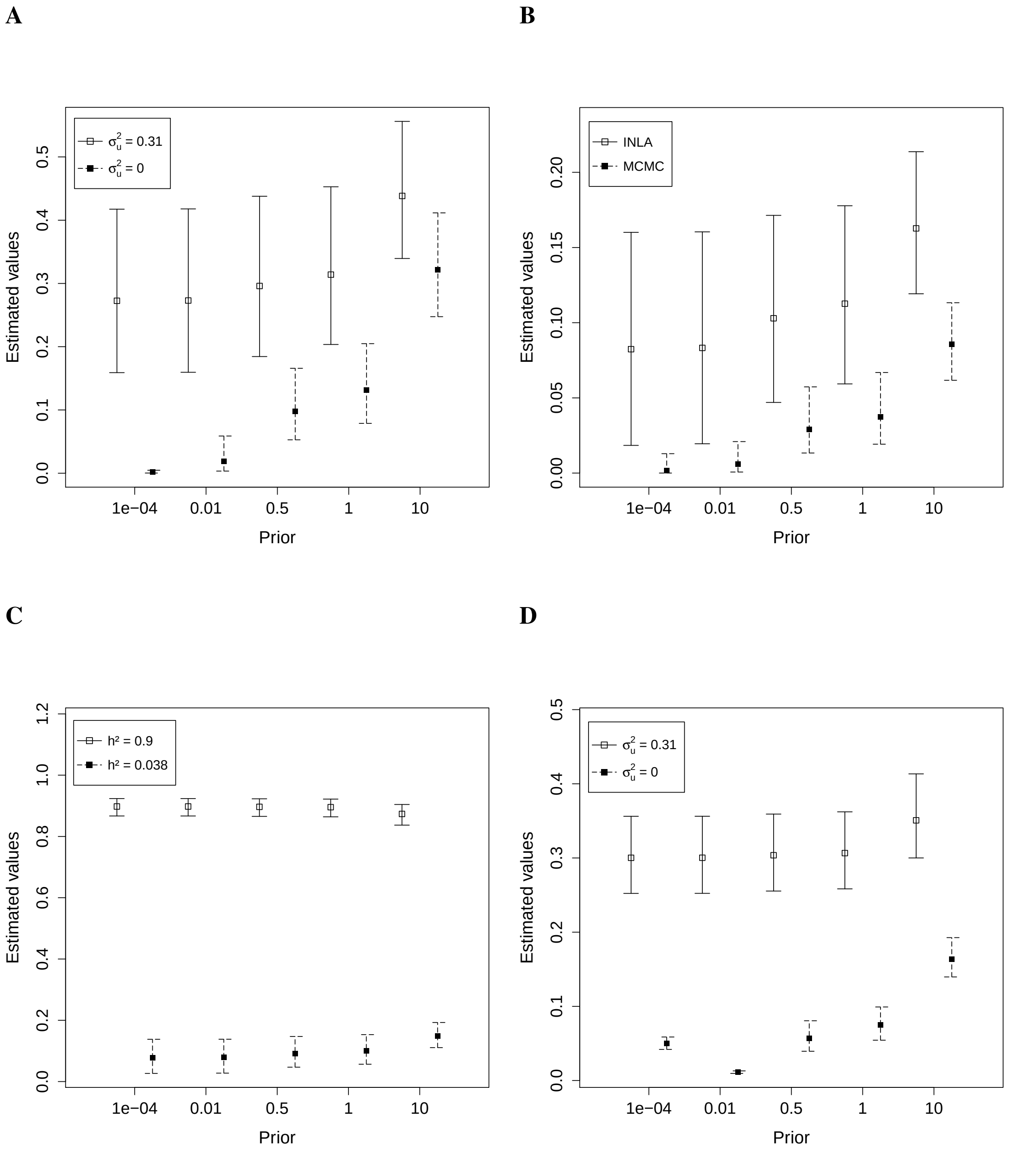

Figure S3 Prior sensitivity analyses for synthetic Gaussian, binary, Binomial, and Poisson case studies. Posterior mean with 95% credible interval for prior InvGamma(a, b), where $a = b$ is equal to 0.0001, 0.01, 0.5, 1 and 10, respectively (note that estimates are shifted relative to the x-axis for clarity). **(A)** Gaussian data for $\sigma_u^2 = 0$ (filled squares, dashed line) and $\sigma_u^2 = 0.31$ (open squares, solid line) for INLA, **(B)** binary data for $h^2 = 0$, INLA (open squares, solid line) and MCMC (filled squares, dashed line), **(C)** Binomial data for $h^2 = 0.9$ (open squares, solid line) and $h^2 = 0.038$ (filled squares, dashed line) for INLA, **(D)** zero-inflated Poisson data for $\sigma_u^2 = 0.31$ (open squares, solid line) and $\sigma_u^2 = 0$ (filled squares, dashed line) for INLA.
