# Supplementary material for: Animal Models and Integrated Nested Laplace Approximations
Source: G3 (Bethesda). 2013 Aug 1;3(8):1241–51. doi: 10.1534/g3.113.006700 (PMC3737164; doi:10.1534/g3.113.006700)
Supplement: Supporting Information [file supp_g3.113.006700_FigureS4.pdf]

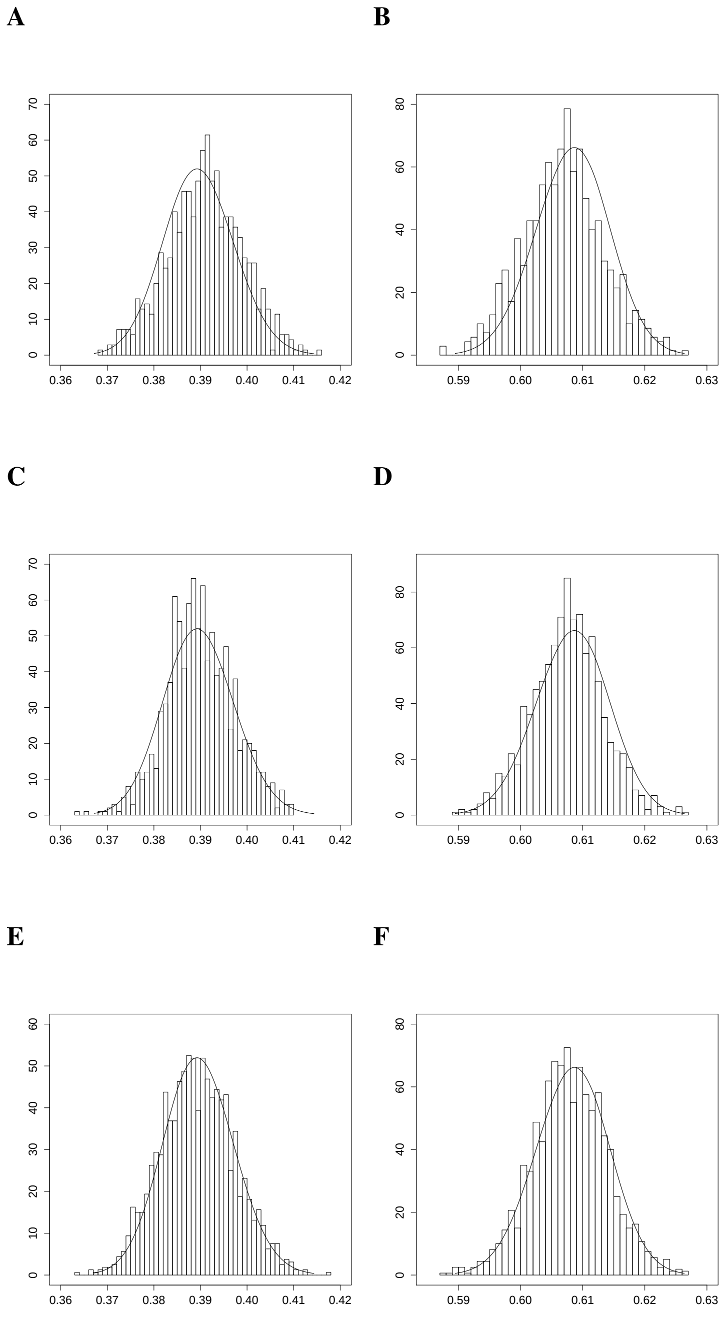

Figure S4 Comparison of INLA and MCMC. INLA (solid line) and MCMC estimates (histogram) for different number of iterations for MCMC for the posterior marginal of σ_u^2 and σ_e^2 for a large synthetic pedigree and simulated dataset of $n_p = 100072$ individuals: 10000 iterations **(A)** σ_u^2 and **(B)** σ_e^2, 100000 iterations **(C)** σ_u^2 and **(D)** σ_e^2, 500000 iterations **(E)** σ_u^2 and **(F)** σ_e^2. INLA used 7.4 minutes and MCMC used 29 minutes, 3.6 hours and 17.9 hours, respectively.
