# Supplementary material for: Animal Models and Integrated Nested Laplace Approximations
Source: G3 (Bethesda). 2013 Aug 1;3(8):1241–51. doi: 10.1534/g3.113.006700 (PMC3737164; doi:10.1534/g3.113.006700)
Supplement: Supporting Information [file supp_g3.113.006700_FigureS5.pdf]

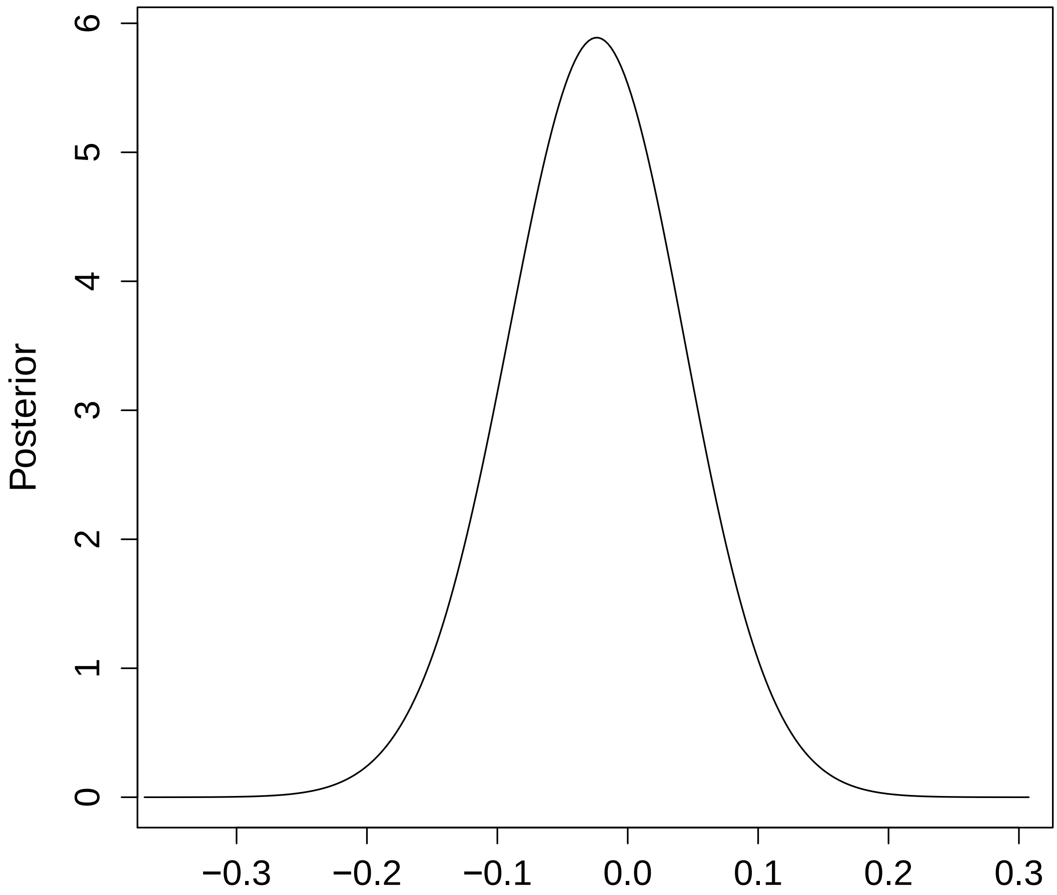

Figure S5 Posterior of difference in mean breeding values for bill depth between cohorts 1993 and 2002 in house sparrows in northern Norway.
