# Supplementary material for: Animal Models and Integrated Nested Laplace Approximations
Source: G3 (Bethesda). 2013 Aug 1;3(8):1241–51. doi: 10.1534/g3.113.006700 (PMC3737164; doi:10.1534/g3.113.006700)
Supplement: Supporting Information [file supp_g3.113.006700_FigureS6.pdf]

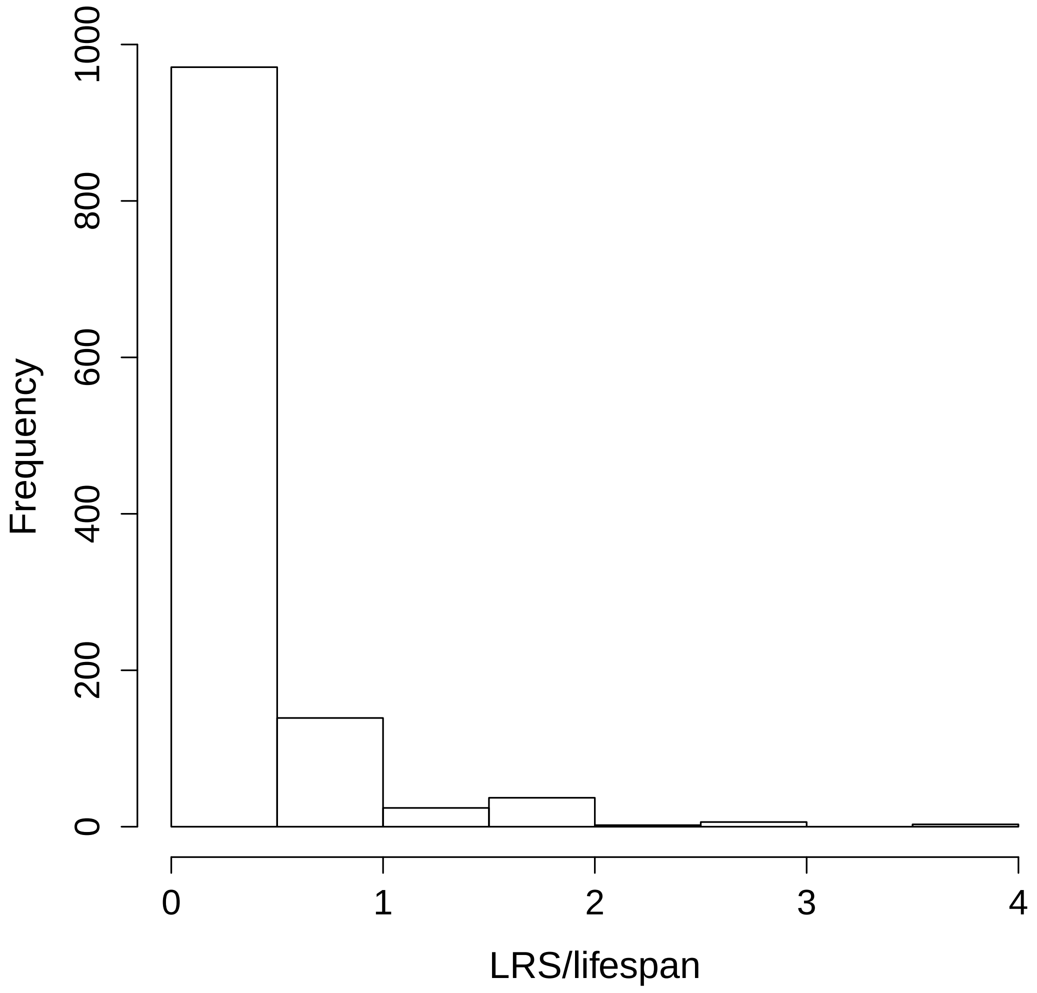


**Figure S6** Histgram showing observed lifetime reproductive sucess (LRS) relative to the lifespan (LRS/lifespan) in house sparrows in northern Norway, indicating a zero-inflated Poisson distribution.
