# Supplementary material for: Animal Models and Integrated Nested Laplace Approximations
Source: G3 (Bethesda). 2013 Aug 1;3(8):1241–51. doi: 10.1534/g3.113.006700 (PMC3737164; doi:10.1534/g3.113.006700)
Supplement: Supporting Information [file supp_g3.113.006700_FileS1.pdf]

File S1

Model formulations for Gaussian animal model

A Gaussian animal model can be formulated in two alternative ways, both fitting the INLA framework.

Model formulation 1 (MF1): Likelihood $y_i|\eta_i \sim \mathcal{N}(\eta_i, \sigma_e^2)$ and latent field $\eta_i = \beta_0 + z_i^T\beta + u_i + \epsilon_i$, where the variance of ϵ is fixed to a small value.

Model formulation 2 (MF2): Likelihood $y_i|\eta_i \sim \mathcal{N}(\eta_i, \sigma_{small}^2)$, i.e. the variance of the likelihood is fixed to a small value, and latent field $\eta_i = \beta_0 + z_i^T\beta + u_i + \epsilon_i$, where the variance of ϵ is σ_e^2. The σ_{small}^2 can be interpreted as measurement uncertainty.

When estimating the narrow sense heritability, h^2, in the Gaussian case, we use model formulation MF2, which out of convenience is parametrized with (σ_u^2, h^2) instead of (σ_u^2, σ_e^2). Further, (σ_u^2, h^2) is given a prior such that it corresponds to the prior of (σ_u^2, σ_e^2), hence, the same prior under two different parametrizations.

The DIC is based on evaluating the likelihood, and is not invariant with respect to parametrization (Spiegelhalter *et al.* 2002). Using model formulation MF2, i.e. a fixed small variance for the likelihood does not work numerically; almost all models get the same DIC to the precision given by INLA. So if DIC needs to be calculated the animal model has to be formulated in an alternative way (in the INLA framework), where the variance of ϵ is fixed to a small value in the latent field, i.e. using MF1. Both model formulations coincide if the same priors are used for the hyper-parameters $(\beta, \sigma_e^2, \sigma_u^2)$, and are latent Gaussian fields with only two non-Gaussian parameters, namely $\boldsymbol{\theta} = (\sigma_u^2, \sigma_e^2)$. For MF1 $\boldsymbol{\epsilon}$ can be omitted from the model. It is included here to be consistent with MF2. Both model formulations have their numerical advantages depending on the aim of the analysis. However, we have to be cautious which model formulation we use depending on the purpose of the analysis.

To summarize, when u_i, $\sum_{i\in C} w_i u_i$, β or σ_u^2 is of interest both MF1 and MF2 might be used. If σ_e^2 or DIC is the aim of the analysis MF1 has to be used, while MF2 with parametrization (σ_e^2, h^2) has to be used if h^2 is of interest. Hence we might have to fit two (INLA) models to get all estimates of interest.

Literature Cited

Spiegelhalter, D. J., N. G. Best, B. P. Carlin, and A. van der Linde, 2002 Bayesian measures of model complexity and fit (with discussion). J. Roy. Stat. Soc. B 64: 583–639.
