# Supplementary material for: Animal Models and Integrated Nested Laplace Approximations
Source: G3 (Bethesda). 2013 Aug 1;3(8):1241–51. doi: 10.1534/g3.113.006700 (PMC3737164; doi:10.1534/g3.113.006700)
Supplement: Supporting Information [file supp_g3.113.006700_FileS2.pdf]

File S2

Prior sensitivity analysis for synthetic datasets

To test for prior sensitivity we do a sensitivity analysis for several synthetic datasets similar to those in **Synthetic case studies section**. The house sparrow pedigree with Gaussian, binary, binomial and Poisson likelihoods are used. Each dataset is analyzed with five different priors for σ_u^2 and, when relevant, σ_ϵ^2; $InvGamma(a, b)$ with $a = b = \{0.0001, 0.01, 0.5, 1, 10\}$. These priors range from uninformative priors to very informative; $InvGamma(10, 10)$ has expected value 1.1 and a standard deviation of 0.37. The results from the sensitivity analyses are visualized in Figure S3.

(A) shows results for two synthetic Gaussian datasets, simulated under model $y_i|\mu_i, \sigma_e^2 \sim \mathcal{N}(\mu_i, \sigma_e^2), \eta_i = \mu_i = \beta_0 + u_i$, with $\beta_0 = 0$ and $\sigma_u^2 + \sigma_\epsilon^2 = 1$ for $i)$ $\sigma_u^2 = 0$ and $ii)$ $\sigma_u^2 = 0.31$. The same missing data structure as in the house sparrow Gaussian case study is imposed giving 1025 individuals in the dataset. Inference is done with INLA. We find that with no heritability ($\sigma_u^2 = 0$) the results are very prior sensitive, while with a heritability of $h^2 = \sigma_u^2 = 0.31$ only the most informative prior changes the inference considerably.

(B) shows results for synthetic binary dataset with observations for all the individuals in the pedigree. The data are simulated from a model with logit link, $\eta_i = \beta_0 + u_i$ with $\beta_0 = 0$ and no genetic component ($\sigma_u^2 = 0$). As we in **Synthetic Binomial case study section** experienced problems using INLA in the binary case, the inference is done with both INLA and MCMC. From the MCMC results we find that the inference is prior sensitive, and also that the systematic errors for INLA are prior sensitive.

(C) shows results for two synthetic binomial datasets. In both datasets the number of trials n_i is as in the house sparrow breeding season success dataset, and also the missing patterns coincide with this. A logit link is used and $\eta_i = \beta_0 + u_i$ with $\beta_0 = 0$. We have a case with high heritability; $i)$ $h^2 = 0.9$ and one with low $ii)$ $h^2 = 0.038$ (or $\sigma_u^2 = 0.13$ as estimated from the breeding season success dataset). Analyses are done using INLA. We find that neither case is very prior sensitive.

(D) shows results for two synthetic zero-inflated Poisson datasets. They are simulated under model $y_i \mid \lambda_i \sim Pois(n_i, \lambda_i), \eta_i = log(\lambda_i) = \beta_0 + u_i$ with $\beta_0 = 0$, with missing pattern as in the house sparrow Poisson case study, and with no heritability ($h^2 = \sigma_u^2 = 0$) and moderate heritability ($\sigma_u^2 = 0.31$). Inference is done with INLA. The results are very prior sensitive for the dataset without heritability, while only the most informative prior gives any considerable difference for the dataset simulated with

$\sigma_u^2 = 0.31.$
