# Supplementary material for: Animal Models and Integrated Nested Laplace Approximations
Source: G3 (Bethesda). 2013 Aug 1;3(8):1241–51. doi: 10.1534/g3.113.006700 (PMC3737164; doi:10.1534/g3.113.006700)
Supplement: Supporting Information [file supp_g3.113.006700_FileS3.pdf]

File S3

R code for synthetic data using the R package `AnimalINLA`

R code for simulating data with same dependency as the real pedigree, where the sparse structure matrix `Cmatrix` is obtained from $\boldsymbol{A}^{-1}$ calculated in the R package `AnimalINLA` (`www.r-inla.org/related-projects/animalinla`).

We simulated data with different values of σ_u^2 =`var.u` and σ_e^2 =`var.e` with the function simulate.breeding.values:

```
Simulation code for breeding value:
##need the package "spam"
install.packages("spam")

inla.complete.Cmatrix <- function(C)
{
    idx = (C$i != C$j)
    return (list(i=c(C$i, C$j[idx]), j=c(C$j, C$i[idx]),
                 values=c(C$values, C$values[idx])))
}
simulate.breeding.values <- function(Cmatrix, varu, nsamples = 1)
{
    library(spam)
    prec = 1/varu
    Comp = inla.complete.Cmatrix(Cmatrix)
    S = spam(x = list(i = Comp$i, j = Comp$j, values =
                                  Comp$values))
    Q = prec * S
    breeding = rmvnorm.prec(nsamples,mu=rep(0, nrow(Q)), Q)
        breeding = as.vector(breeding)
```

```
}

##define the sparse-matrix from the relationship matrix
##computed in compute.Ainverse(), used in simulate.breeding.values()
Cmatrix = list(i= xx$Ainverse[,1],j = xx$Ainverse[,2], values =xx$Ainverse[,3])
```

Synthetic Gaussian case study

```
library(AnimalINLA)
data(sparrowpedigree)

##Run AnimalINLA
xx=compute.Ainverse(sparrowpedigree)

##number of individuals in the pedigree
Nbird = dim(sparrowpedigree)[1]
## choose the values of the hyperparameters
var.u = 0.6
var.e = 0.4

## simulate the breeding values and the environemental effect
breeding = simulate.breeding.values(Cmatrix, var.u)
env = rnorm(Nbird, mean = 0, sd = sqrt(var.e))

## compute the trait
trait =  breeding + env

## make the data frame
data = data.frame(y=trait,u=1:Nbird)
```

```
##Run AnimalINLA
gauss=animal.inla(response=y, genetic=c("u"),
                  Ainverse =sparseMatrix(i=xx$Ainverse[,1],
                  j=xx$Ainverse[,2],x=xx$Ainverse[,3]),
                  data=data, type.data="gaussian",
                  dic=TRUE,sigma.e=TRUE)

##hyperparameteres
gauss$summary.hyperparam
```

Synthetic Binomial case study

```
library(AnimalINLA)
data(sparrowpedigree)

##need the package "boot"
install.packages("boot")
library(boot)

## numbers of individuals in the pedigree
Nbird = dim(pedigree)[1]

## set the value for the hyperparameter, where beta0 is the intercept
var.u = 0.3
beta0 = 1

## set the number of trials
Ntrials = sample(1:9, 3574 , replace=T)

## simulate breeding values
```

```
breeding = simulate.breeding.values(Cmatrix, var.u)
eta =  beta0 + breeding
p = inv.logit(eta)

## simulate the trait
trait = rbinom(Nbird, Ntrials, p)

data = data.frame(y = trait,u = 1:Nbird,
                  Ntrial = Ntrials)

##Run AnimalINLA
xx=compute.Ainverse(sparrowpedigree)

bin=animal.inla(response=y, genetic=c("u"),
                Ntrials = Ntrial,
                Ainverse =sparseMatrix(i=xx$Ainverse[,1],
                j=xx$Ainverse[,2],x=xx$Ainverse[,3]),
                data=data,type.data="binomial",
                dic=TRUE)

##hyperparameteres
bin$summary.hyperparam
```

Synthetic Poisson case study

```
library(AnimalINLA)
data(sparrowpedigree)

##number of individuals in the pedigree
Nbird = dim(sparrowpedigree)[1]
```

```
## choose the values of the hyperparameters
var.u =  0.7
beta0 = 1

##Run AnimalINLA
breeding = simulate.breeding.values(Cmatrix, var.u)

## compute the trait
eta = beta0 + breeding
lambda=exp(eta)
trait=rpois(Nbird,lambda)

## make the data frame
data = data.frame(y=trait,u=1:Nbird,n=rep(1,Nbird))

##Run AnimalINLA
xx=compute.Ainverse(sparrowpedigree)

pois=animal.inla(response="y", genetic=c("u"),
                 Ainverse =sparseMatrix(i=xx$Ainverse[,1],
                 j=xx$Ainverse[,2],x=xx$Ainverse[,3]),
                 E=n,data=data,type.data="poisson",dic=TRUE)

##hyperparameters
pois$summary.hyperparam
```
