# Supplementary material for: Animal Models and Integrated Nested Laplace Approximations
Source: G3 (Bethesda). 2013 Aug 1;3(8):1241–51. doi: 10.1534/g3.113.006700 (PMC3737164; doi:10.1534/g3.113.006700)
Supplement: Supporting Information [file supp_g3.113.006700_FileS4.pdf]

File S4

R code for random effects in INLA

Including individual as a independent random effect in the latent field is implemented the same way in INLA for all case studies in house sparrow population (Gaussian, binomial and Poisson). Note that in the Gaussian case study we have repeated measurements, i.e. possible several observation for each individual random effect, while in the binomial and Poisson cases there are only one observation for each individual. For the simulated datasets in `AnimalINLA` (only one measurement for each individual);

```
library(AnimalINLA)
library(INLA)
library(Matrix)

data(sparrowpedigree)
xx = compute.Ainverse(sparrowpedigree)
Ainv = xx$Ainverse
map  = xx$map
Cmatrix = sparseMatrix(i=Ainv[,1],j=Ainv[,2],x=Ainv[,3])
```

Gaussian case study:

```
data(sparrowGaussian)
Ndata = dim(sparrowGaussian)[1]

## Mapping the same index number for "Individual" as in Ainv
## The IndexA column is the index in the A inverse matrix
sparrowGaussian$IndexA = rep(0,Ndata)
for(i in 1:Ndata)
  sparrowGaussian$IndexA[i] = which(map[,1]==sparrowGaussian$Individual[i])

#Including an extra column for individual effect
sparrowGaussian$IndexA.2=sparrowGaussian$IndexA
```

```
formula = y ~ f(IndexA,model="generic0", Cmatrix=Cmatrix,
    constr=TRUE,param = c(0.5, 0.5)) +
                      f(IndexA.2,model="iid",param = c(1,0.001),
                      constr=TRUE)
```

y in $formula$ is the trait, i.e bill depth in the case study, *IndexA* and *IndexA.2* is the individuals in the data (these have to be given different names) where *IndexA* is the additive genetic effect and *IndexA.2* is the individual random effect.

the likelihood is implemented in the inla call;

```
model = inla(formula=formula, family="gaussian",
         data=sparrowGaussian,
         control.family=list(hyper = list(theta =
         list(param = c(0.5, 0.5), fixed = FALSE))),
 only.hyperparam =FALSE,control.compute=list(dic=T))

summary(model)
#Example finding the posterior marginal distribution and mean (95% CI)
#for additive genetic variance and individual random variance
sigma.IndexA = inla.marginal.transform(function(x) 1/x,
             model$marginals.hyperpar$"Precision for IndexA")
e.IndexA=inla.expectation(function(x) x, sigma.IndexA)
ci.IndexA=inla.qmarginal(c(0.025, 0.975), sigma.IndexA)

#and posterior marginal distribution and mean (95% CI)
#for individual random variance
sigma.IndexA.2 = inla.marginal.transform(function(x) 1/x,
                model$marginals.hyperpar$"Precision for IndexA.2")
e.IndexA.2=inla.expectation(function(x) x, sigma.IndexA.2)
ci.IndexA.2=inla.qmarginal(c(0.025, 0.975), sigma.IndexA.2)
```

Binomial case study:

```
data(sparrowBinomial)

Ndata = dim(sparrowBinomial)[1]

## Mapping the same index number for "Individual" as in Ainv
## The IndexA column is the index in the A inverse matrix
sparrowBinomial$IndexA = rep(0,Ndata)
for(i in 1:Ndata)
  sparrowBinomial$IndexA[i] = which(map[,1]==sparrowBinomial$Individual[i])

#Including an extra column for individual effect
sparrowBinomial$IndexA.2=sparrowBinomial$IndexA

formula = y ~ f(IndexA,model="generic0", Cmatrix=Cmatrix,
    constr=TRUE,param = c(0.5, 0.5)) +
                      f(IndexA.2,model="iid",param = c(1,0.001),
                      constr=TRUE)
```

y in $formula$ is the trait, i.e number of years individuals produced at least one recruit in the case study, *IndexA* and *IndexA.2* is the individuals in the data (these have to be given different names) where *IndexA* is the additive genetic effect and *IndexA.2* is the individual random effect.

The likelihood is implemented in the inla call;

```
model = inla(formula=formula , family="binomial", data=sparrowBinomial,
 Ntrials=Ntrial,
         only.hyperparam = FALSE,control.compute=list(dic=T))
```

Ntrial is the number of trials, i.e the number of breeding seasons individuals were alive during the study period.

```
summary(model)
#Example finding the posterior marginal distribution and mean (95% CI) for
#additive genetic variance and individual random variance
sigma.IndexA = inla.marginal.transform(function(x) 1/x,
               model$marginals.hyperpar$"Precision for IndexA")
e.IndexA=inla.expectation(function(x) x, sigma.IndexA)
ci.IndexA=inla.qmarginal(c(0.025, 0.975), sigma.IndexA)

#and posterior marginal distribution and mean (95% CI)
#for individual random variance
sigma.IndexA.2 = inla.marginal.transform(function(x) 1/x,
                model$marginals.hyperpar$"Precision for IndexA.2")
e.IndexA.2=inla.expectation(function(x) x, sigma.IndexA.2)
ci.IndexA.2=inla.qmarginal(c(0.025, 0.975), sigma.IndexA.2)
```

Poisson case study:

```
data(sparrowPoisson)

Ndata = dim(sparrowPoisson)[1]

## Mapping the same index number for "Individual" as in Ainv
## The IndexA column is the index in the A inverse matrix
sparrowPoisson$IndexA = rep(0,Ndata)
for(i in 1:Ndata)
sparrowPoisson$IndexA[i] = which(map[,1]==sparrowPoisson$Individual[i])

#Including an extra column for individual effect
```

```
sparrowPoisson$IndexA.2=sparrowPoisson$IndexA

formula = y ~ f(IndexA,model="generic0", Cmatrix=Cmatrix,
    constr=TRUE,param = c(0.5, 0.5)) +
                      f(IndexA.2,model="iid",param = c(1,0.001),
                      constr=TRUE)
```

y in $formula$ is the trait, i.e total number of recruits individuals produced in the study period in the case study, *IndexA* and *IndexA.2* is the individuals in the data (these have to be given different names) where *IndexA* is the additive genetic effect and *IndexA.2* is the individual random effect.

The likelihood is implemented in the inla call;

```
model = inla(formula=formula,
              family="zeroinflatedpoisson1",
              #family="poisson" ,
              data=sparrowPoisson,
              E=n,
              only.hyperparam = FALSE,
              control.compute=list(dic=TRUE))
```

E is the exposure, i.e. the number of breeding seasons individuals were alive during the study period in the case study.

```
summary(model)
#Example finding the posterior marginal distribution and mean (95% CI)
#for additive genetic variance and individual random variance
sigma.IndexA = inla.marginal.transform(function(x) 1/x,
                model$marginals.hyperpar$"Precision for IndexA")
e.IndexA=inla.expectation(function(x) x, sigma.IndexA)
ci.IndexA=inla.qmarginal(c(0.025, 0.975), sigma.IndexA)

#and posterior marginal distribution and mean (95% CI)
```

```
#for individual random variance
sigma.IndexA.2 = inla.marginal.transform(function(x) 1/x,
               model$marginals.hyperpar$"Precision for IndexA.2")
e.IndexA.2=inla.expectation(function(x) x, sigma.IndexA.2)
ci.IndexA.2=inla.qmarginal(c(0.025, 0.975), sigma.IndexA.2)
```
