# Supplementary material for: Animal Models and Integrated Nested Laplace Approximations
Source: G3 (Bethesda). 2013 Aug 1;3(8):1241–51. doi: 10.1534/g3.113.006700 (PMC3737164; doi:10.1534/g3.113.006700)
Supplement: Supporting Information [file supp_g3.113.006700_TableS1.pdf]

Table S1 Inference from INLA for synthetic Poisson data. Simulated under model $\mathbf{y_i} \mid \lambda_\mathbf{i} \sim \mathbf{Pois}(\mathbf{n_i}, \lambda_\mathbf{i})$, $\eta_\mathbf{i} = \mathbf{log}(\lambda_\mathbf{i}) = \beta_\mathbf{0} + \mathbf{u_i}$, with $\beta_\mathbf{0} = \mathbf{0}$, levels of σ_u^2 ranging from 0 to 1, and missing pattern similar to the house sparrow Poisson case study. $\hat{\sigma}_\mathbf{u}^\mathbf{2}$ is the posterior mean additive genetic variance with standard deviations (sd), and 95% credible interval (CI).

$\boldsymbol{\sigma_u^2}$	$\hat{\sigma}_u^2$ (sd)	95% CI
**0**	0.08 (0.02)	(0.05,0.13)
**0.05**	0.09 (0.02)	(0.05,0.13)
**0.1**	0.12 (0.03)	(0.07,0.18)
**0.15**	0.14 (0.03)	(0.09,0.21)
**0.2**	0.20 (0.04)	(0.13,0.27)
**0.3**	0.33 (0.05)	(0.25,0.43)
**0.4**	0.43 (0.05)	(0.33,0.54)
**0.5**	0.53 (0.06)	(0.43,0.65)
**0.6**	0.60 (0.06)	(0.49,0.73)
**0.7**	0.68 (0.06)	(0.56,0.81)
**0.8**	0.84 (0.08)	(0.69,1.00)
**0.9**	0.91 (0.08)	(0.77, 1.08)
**1**	0.99 (0.09)	(0.83,1.17)
